# Shuangyu Tiaozhi decoction alleviates non-alcoholic fatty liver disease by improving lipid deposition, insulin resistance, and inflammation *in vitro* and *in vivo*

**DOI:** 10.3389/fphar.2022.1016745

**Published:** 2022-11-23

**Authors:** Guoliang Yin, Hongyi Liang, Wenxiu Sun, Shizhao Zhang, Yanan Feng, Pengpeng Liang, Suwen Chen, Xiangyi Liu, Wenchao Pan, Fengxia Zhang

**Affiliations:** ^1^ The First Clinical Medical School, Shandong University of Traditional Chinese Medicine, Jinan, China; ^2^ Department of Nursing, Taishan Vocational College of Nursing, Taian, China; ^3^ Department of Neurology, Affiliated Hospital of Shandong University of Traditional Chinese Medicine, Jinan, China

**Keywords:** Shuangyu Tiaozhi decoction, non-alcoholic fatty liver disease, lipid deposition, insulin resistance, inflammation

## Abstract

Non-alcoholic fatty liver disease (NAFLD) is one of the most common chronic liver diseases worldwide. Our previous studies have found that Shuangyu Tiaozhi Decoction (SYTZD) could produce an improvement in NAFLD-related indicators, but the underlying mechanism associated with this improvement remains unclear. The study aimed to investigate the potential mechanism of SYTZD against NAFLD through network pharmacology and experimental verification. The components of SYTZD and SYTZD drug containing serum were analyzed using ultra-performance liquid chromatography to quadrupole/time-of-flight mass spectrometry (UPLC-Q/TOF-MS). Active components and targets of SYTZD were screened by the traditional Chinese medical systems pharmacology (TCMSP) and encyclopedia of traditional Chinese medicine (ETCM) databases. NAFLD-related targets were collected from the GeneCards and DisGeNET databases. The component-disease targets were mapped to identify the common targets of SYTZD against NAFLD. Protein–protein interaction (PPI) network of the common targets was constructed for selecting the core targets. Kyoto Encyclopedia of Genes and Genomes (KEGG) pathway analysis of the core targets was performed using the database for annotation, visualization, and integrated discovery (DAVID) database. Furthermore, animal and cell models were constructed for validating the predictions of network pharmacology. Lipid accumulation, liver histopathology, insulin resistance, and core gene expression were measured by oil red O staining, hematoxylin and eosin staining, insulin tolerance test, real-time quantitative polymerase chain reaction, and Western blotting, respectively. Two components and 22 targets of SYTZD against NAFLD were identified by UPLC-Q/TOF-MS and relevant databases. PPI analysis found that ESR1, FASN, mTOR, HIF-1α, VEGFA, and GSK-3β might be the core targets of SYTZD against NAFLD, which were mainly enriched in the thyroid hormone pathway, insulin resistance pathway, HIF-1 pathway, mTOR pathway, and AMPK pathway. Experimental results revealed that SYTZD might exert multiple anti-NAFLD mechanisms, including improvements in lipid deposition, inflammation, and insulin resistance. SYTZD treatment led to decreases in the lipid profiles, hepatic enzyme levels, inflammatory cytokines, and homeostatic model assessment for insulin resistance (HOMA-IR). SYTZD treatment affected relative mRNA and protein levels associated with various pathways. Our findings reveal that SYTZD could alleviate NAFLD through a multi-component, multi-target, and multi-pathway mechanism of action.

## Introduction

Non-alcoholic fatty liver disease (NAFLD) is one of the most common chronic liver diseases worldwide and is an important cause of increased morbidity and mortality from liver-related diseases (Chalasani et al., 2018; Loomba et al., 2021). NAFLD is closely associated with obesity, dyslipidemia, metabolic syndrome, and type 2 diabetes mellitus (Polyzos et al., 2019) and can progress from simple steatosis to hepatitis and even cirrhosis and liver cancer (Wang and Malhi, 2018). Recently, the prevalence of NAFLD has been increasing worldwide with more than 25% of the adult population suffers from this disease (Cotter and Rinella, 2020). The proportion of obese and overweight people in China has increased to 38.5%, and the prevalence of NAFLD reached 29.8% in 2019 (Zhou et al., 2019; Kanwal et al., 2021). Therefore, it is of great practical importance to study ways to effectively intervene in NAFLD.

The exact pathogenesis of NAFLD is still unclear, and the “2-hit” theory is universally acknowledged (Fang et al., 2018; Bessone et al., 2019; Marchisello et al., 2019). The “first hit,” also known as steatosis, is caused by disorders of lipid metabolism due to fat accumulation in the liver and insulin resistance (Masarone et al., 2018). NAFLD is further exacerbated by fat accumulation, accompanied by an inflammation, oxidative stress and apoptosis, which creates a “second hit” to the liver (Flisiak-Jackiewicz and Lebensztejn, 2019). NAFLD treatment includes lifestyle interventions, medication, and surgery (Sheka et al., 2020). However, as NAFLD is associated with metabolic abnormalities, single-target therapy with Western drugs does not achieve the desired effect (Mundi et al., 2020). Currently, no drugs that have been approved by the United States Food and Drug Administration (FDA) specifically for the treatment of NAFLD are available (Friedman et al., 2018). Therefore, it is urgent to find safe and effective agents for the treatment of NAFLD and its complications.

Traditional Chinese medicine (TCM) has achieved remarkable efficacy for the treatment of NAFLD in the clinical (Yan et al., 2020; Lu et al., 2021). According to the principles of TCM, NAFLD is located in the liver, which is closely related to the spleen and kidney, and the pathogenesis of NAFLD is considered liver dysfunction and spleen deconditioning, and sputum dampness and blood stasis are important pathological factors associated with NAFLD (Dai et al., 2021). Therefore, the treatment of strengthening the spleen, relieving dampness, and resolving turbidity is an important aspect. Shuangyu Tiaozhi Decoction (SYTZD) consists of two botanical drugs, *Dioscorea oppositifolia L.* [Dioscoreaceae; Dioscoreae rhizoma] (Shanyao in Chinese) and *Dioscorea septemloba Thunb.* [Dioscoreaceae; Dioscoreae spongiosae rhizoma] (Mianbixie in Chinese). In the formula, *Dioscorea oppositifolia L.* is mainly used and is paired with *Dioscorea septemloba Thunb.* to invigorate the spleen and replenish qi thus eliminating phlegm and removing turbidity. *Dioscorea oppositifolia L.* is both a common food and Chinese herbal medicine, and it has been reported that it exerts effects on reducing lipid synthesis, modulating the gut microbiota, and inhibiting fat accumulation (Dissanayake et al., 2021; Zhou et al., 2022). Additionally, our previous studies demonstrated that SYTZD could produce improvements in the NAFLD-related indicators, including lipid deposition and hepatocyte steatosis (Shi et al., 2019). However, these studies were conducted on a single mechanism and failed to systematically analyze the multi-target- and multi-pathway-associated mechanisms of action.

**FIGURE 1:**
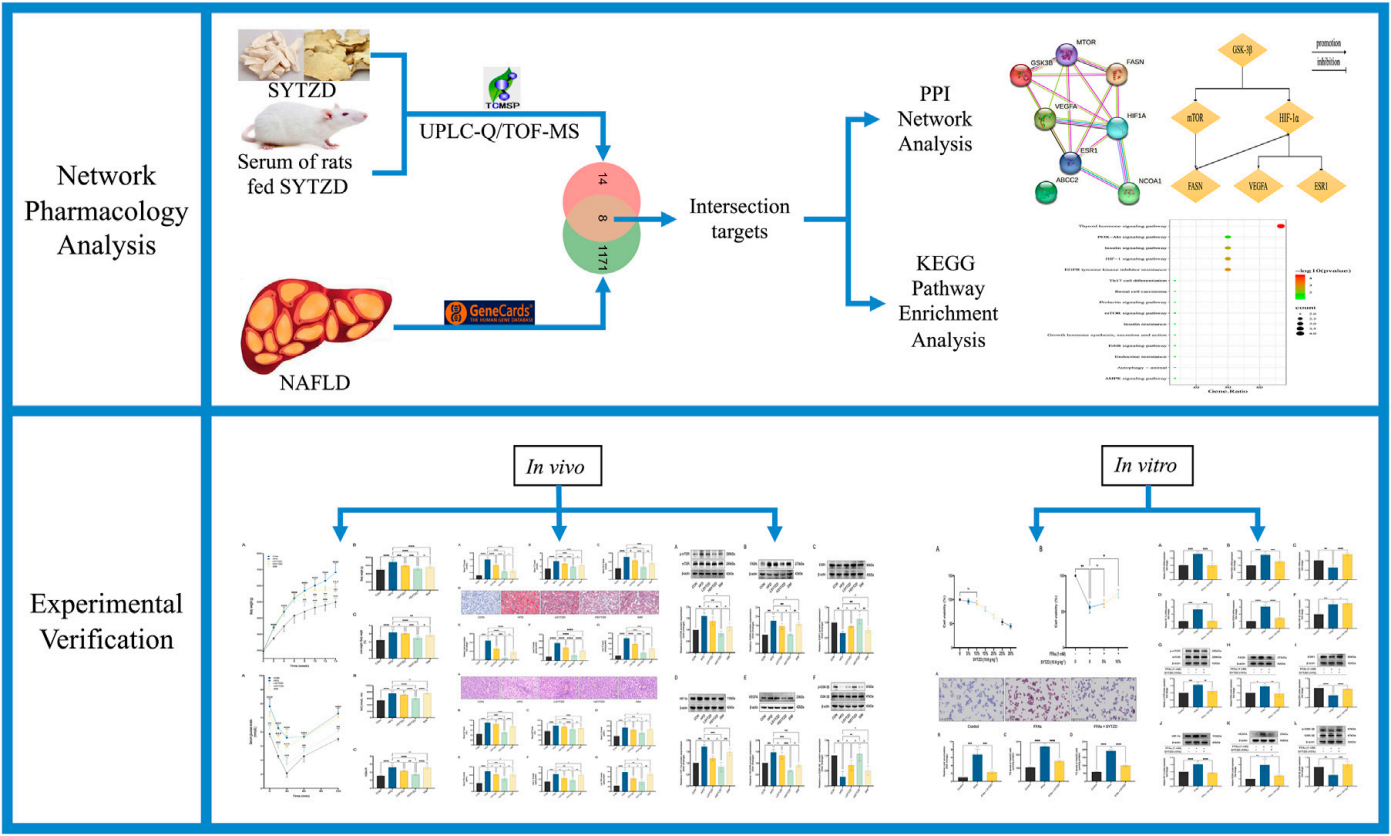
Flow diagram of this study.

Network pharmacology is based on the theory of systems biology and multidirectional pharmacology (Zhou et al., 2020). The systemic characteristics of network pharmacology are compatible with the holistic view of TCM (Huang et al., 2021a), which is promising for the interpretation of the pharmacological mechanism of TCM from the perspective of multi-component, multi-target, and multi-pathway systems (Luoting et al., 2020). This study aimed to explore the potential therapeutic targets and mechanism of action of SYTZD for NAFLD treatment based on network pharmacology and experimental validation (Figure 1). Our findings provide scientific evidence for supporting the clinical application of SYTZD for NAFLD treatment and provides a reliable reference for exploring the pharmacological mechanisms of SYTZD.

## Materials and methods

### Preparation of SYTZD

The composition of SYTZD included 60 g of *Dioscorea oppositifolia L.* and 18 g of *Dioscorea septemloba Thunb*. The botanical drugs were provided by the herbal pharmacy of the Affiliated Hospital of Shandong University of Traditional Chinese Medicine and were identified by Professor Feng Li of Pharmacy College, Shandong University of Traditional Chinese Medicine. The botanical drugs were steeped in 600 ml of water for 30 min. After boiling at 100°C, the mixture was heated for 20 additional min, and the decoction was separated. Another 400 ml of water was added, and the second decoction was prepared for 15 min according to the same method. The two decoctions were combined and filtered with gauze. The combined decoction was concentrated to 50 ml by boiling water under a rotary evaporator (N-1300, Shanghai Ailang Instrument Co., Ltd., Shanghai, China). Finally, 1.56 g/ml of drug decoction was prepared and stored in the refrigerator at 4°C.

### Preparation of SYTZD drug containing serum

Six-week-old Sprague-Dawley rats, weighing between 180 and 220 g were purchased from the Vital River Laboratory Animal Technology Co. Ltd. (Beijing, China) with a certificate number of No. SCXK (Jing) 2016-0011. All animals were housed in an environment with temperature of 23 ± 2°C, humidity of 50% ± 10%, and 12 h light/12 h dark cycle. Rats had free access to water and food. After adaptive feeding for 1 week, 20 rats were randomly divided into two groups (10 per group): (Chalasani et al., 2018) control and (Loomba et al., 2021) SYTZD. Rats in the SYTZD group were given 15.6 g/kg/d of SYTZD by gavage once a day for 7 days, and the dose was referenced to the equivalent body surface area conversion method (Liu et al., 2020). Rats in the control group were given the same volume of saline (5 ml/kg) *via* gavage. After fasting for 12 h, rats in SYTZD group were given SYTZD by gavage for 1–1.5 h, and all rats were anesthetized by sodium pentobarbital (40 mg/kg) intraperitoneally (Luo et al., 2021). Blood was then collected from the abdominal aorta and centrifuged at 3,000 rpm for 15 min. After centrifugation, the serum from the same group was combined and inactivated at 56°C for 30 min. The serum was filtered with 0.22-µm filters, aliquoted, and stored at −20°C (Djigo et al., 2019).

### UPLC-Q/TOF-MS for component analysis

The extract of SYTZD was diluted with methanol, filtered through a 0.22 µm filter, and then subjected to ultra-performance liquid chromatography to quadrupole/time-of-flight mass spectrometry (UPLC-Q/TOF-MS) analysis to characterize the chemical composition. UPLC-Q/TOF-MS analysis was achieved on an UHPLC LC-30A system (SHIMADZU, China) coupled with a Q/TOF 6600 mass spectrometry (AB SCIEX, MA, United States). The extract was separated on an ACQUITY UPLC HSS T3 column (100 mm × 2.1 mm, 1.8 µm, Waters, MA, United States). The mobile phase was 0.1% formic acid water (A) and 0.1% formic acid in acetonitrile (B). The elution gradient of phase B was depicted as follows: 5% up to 90% (0–11 min), maintained at 90% (11–12 min), 90% down to 5% (12–12.5 min), maintained at 5% (12.5–15 min). The flow rate of the mobile phase was 0.3 ml/min, the column temperature was 40°C, and the injection volume was 5 µl. The mass spectrometer analysis was carried out with TurboIon Spray ion source and electrospray ionization (ESI) positive and negative ion scanning mode, and optimized parameters were as follows: Ion Source Gas1 (Gas1): 55, Ion Source Gas2 (Gas2): 55, Curtain gas (CUR): 35, source temperature: 550°C, IonSapary Voltage Flowing (ISVF): 5,500 V/−4,500 V; TOF MS scan m/z range: 50–1,500 Da, production scan m/z range: 25–1,000 Da, TOF MS scan accumulation time 0.25 s/spectra, product ion scan accumulation time 0.035 s/spectra; Secondary mass spectrometry uses information-dependent acquisition (IDA) and high sensitivity mode, Declustering potential (DP): ±60 V (positive and negative mode), Collision Energy was 30 ± 15 eV. The mass spectrometry data were collected and processed by SCIEX OS software. The SCIEX OS software contains multiple confidence criteria, including quality accuracy, retention time, isotopes, and matching use of compound libraries. In this experiment, TCM MS/MS Library (including secondary data of more than 1,000 Chinese herbal medicines) can be searched according to the first-order accurate mass number, isotope distribution ratio and MS/MS of the compounds, and then the screening of the target compounds can be completed. Rat serum was also analyzed using the same conditions to investigate the constituents in rat serum.

### Collection of active ingredients and targets of SYTZD

Using “Shanyao” and “Mianbixie” as keywords, the TCM systems pharmacology database and analysis platform (TCMSP, https://old.tcmsp-e.com/tcmsp.php) database was used to screen the active compounds of SYTZD. The screening criteria were set as oral availability (OB) ≥ 30% and drug-likeness (DL) ≥ 0.18 (Zhang et al., 2021). In addition, the TCMSP database and encyclopedia of traditional Chinese medicine (ETCM, http://www.tcmip.cn/ETCM/index.php/Home/) database were used to obtain the targets of the screened components. The results were then combined with the corresponding literature search results (Jayaraman et al., 2020; Wang et al., 2021a). Finally, the component targets were integrated into standard gene symbol using the Uniport platform (https://www.uniprot.org/) (jing et al., 2022).

### Collection of NAFLD-related targets

With “nonalcoholic fatty liver disease” as the keyword, the GeneCards (http://www.genecards.org/) database and DisGeNET (https://www.disgenet.org/) databases were used to collect NAFLD-related targets (Cao et al., 2021). The screened component targets and disease targets were mapped using the Venny platform (http://bioinformatics.psb.ugent.be/webtools/Venn/) to identify the candidate targets of SYTZD for NAFLD treatment (Wang et al., 2021a).

### PPI network construction and core target screening

The “component-disease” common targets were entered into the STRING platform (https://stringdb.org/), and a protein–protein interaction (PPI) visual network was constructed. The species was set as “*Homo sapiens*,” the minimum interaction threshold was set as 0.4, and the other parameters remained at the default values. The tsv file of the PPI network was then imported into Cytoscape 3.7.2 software, the gene cluster analysis was performed using the MCODE plug-in for obtaining the gene cluster, and the core gene network was constructed (Wang et al., 2022).

### KEGG pathway enrichment analysis

The core targets were submitted to the DAVID database (https://david.ncifcrf.gov/) for performing Kyoto Encyclopedia of Genes and Genomes (KEGG) pathway enrichment analysis (Szklarczyk et al., 2019). The species was set as “*Homo sapiens*.” With a screening condition of *p* < 0.05, the pathways of the core targets were obtained, and the results were analyzed for determining the top 15 of pathways. Finally, the results were visualized as bubble plots using the bioinformatics platform (http://bioinformatics.com.cn/).

### Animal modeling and treatment

High-fat diets (HFD) were provided from Xiaoshu Youtai Biotechnology Co., Ltd. (Beijing, China), and the formulations consisted of Lard 10%, egg yolk powder 10%, cholesterol 1%, and basal diets 93.2% (protein 20.8%, fat 34.9%, carbohydrate 44.3%). After adaptive feeding with normal diets for 1 week, 40 male rats were randomly divided into five groups (eight per group): (Chalasani et al., 2018) CON, (Loomba et al., 2021) HFD, (Polyzos et al., 2019) LSYTZD, (Wang and Malhi, 2018) HSYTZD, and (Cotter and Rinella, 2020) SIM. Rats in the CON group were fed normal diets, and the other rats were fed HFD for 6 weeks to construct NAFLD rat models. Among the five groups, rats in the LSYTZD group were given 7.8 g/kg/d of SYTZD, rats in the HSYTZD group were given 15.6 g/kg/d of SYTZD, rats in the SIM group were given 4 mg/kg/d of simvastatin, and rats in the CON and the HFD groups were given an equivalent amount of normal saline (5 ml/kg). All rats were treated *via* gavage once a day for 8 weeks. During administration, rats in the CON group continuedly fed normal diets, and rats in the other groups continuedly fed HFD. All rats were weighed every fortnight. All experiments were conducted in accordance with the Guide for the Care and Use of Laboratory Animals and approved by the Animal Ethics Committee of Shandong University of Traditional Chinese Medicine (approval number: 2021-94).

### Insulin tolerance test

Rats were fasted for 12 h before the last dose, blood was taken from tail, and fasting blood glucose (FBG) was measured. All rats were intraperitoneally injected with insulin reagent (1 U/kg, Novo Nordisk Pharmaceutical Co., Ltd., Tianjin, China). Blood glucose was measured at 15, 30, 60, 90, and 120 min post-injection, insulin tolerance time-dependent curves were plotted, and the area under the curve (AUC) was calculated (Bellantuono et al., 2020).

### Serum and tissue sample collection

After fasting for 12 h after the last dose, all rats were anaesthetized with sodium pentobarbital (40 mg/kg) intraperitoneally, blood was drawn from abdominal aorta, left at room temperature for 1 h and centrifuged at 3,000 rpm for 15 min at 4°C for serum collection. The serum was stored at −20°C for subsequent analysis. Liver tissues were rapidly collected after blood was taken, rinsed with frozen saline, weighed, and stored at −80°C for the subsequent experiments.

### Insulin contents assay

The levels of fasting insulin (FINS) were measured using insulin enzyme-linked immunosorbent assay (ELISA) Kit (Elabscience Biotechnology Co., Ltd., Wuhan, China). The homeostasis model assessment insulin resistance (HOMA-IR) was used to estimate the levels of insulin resistance. HOMA-IR was calculated as follows: HOMA-IR = FBG × FINS/22.5 (Yu et al., 2020).

### Lipid contents and hepatocyte metabolic enzyme assay

The levels of serum total cholesterol (TC), triglycerides (TG), low-density lipoprotein cholesterol (LDL-C), alanine aminotransferase (ALT), and aspartate aminotransferase (AST) in rats were measured in the department of Clinical Laboratories of the Affiliated Hospital of Shandong University of Traditional Chinese Medicine (Shandong, China). The TG and TC contents of HepG2 cells and rats were determined by Triglyceride Assay Kit and Total Cholesterol Assay Kit according to the manufacturer’s instructions (Applygen, Beijing, China), respectively.

### Inflammatory factor assay

The levels of serum interleukin 1 beta (IL-1β) and tumor necrosis alpha (TNF-α) in rats were detected using ELISA kits (Applygen, Beijing, China) according to the manufacturer’s instructions. The levels of liver IL-1β and TNF-α in rats were detected using ELISA kits (Jiangsu MEIMIAN Industrial Co., Ltd., Jiangsu, China) according to the manufacturer’s instructions.

### Oil red O staining

Liver lipid deposition was measured using oil red O staining kit (Solarbio Biotechnology, Beijing, China). Briefly, oil red O working solution was prepared by mixing the oil red O stock solution with distilled water (oil red O: distilled water = 3: 2), incubating at room temperature for 10 min, and filtered with filter paper. The 8 µm sections of tissue were attached to slides and fixed with formaldehyde-calcium for 10 min. The sections were washed with 60% isopropanol for 30 s, stained with oil red O working solution for 10 min, and re-stained with hematoxylin for 5 min. The sections were sealed with glycerol gelatin. Finally, the sections were observed and photographed under a digital section scanner (3D HISTECH, DANJIER Electronic Co., Ltd., Jinan, China).

Intracellular lipid accumulation was estimated using cell specific oil red O staining kit (Solarbio Biotechnology, Beijing, China). HepG2 cells were seeded into 6-well plates at a density of 5 × 10^4^, adherent overnight, followed by treated with 10% SYTZD drug containing serum for 24 h. Oil red O staining was carried out according to the manufacturer’s instructions (Zhou et al., 2021). The stained cells were observed and photographed under a digital section scanner. Finally, the stained area was quantified using ImageJ software.

### H&E staining

Liver histopathological morphology was examined using a hematoxylin and eosin (H&E) staining kit (Solarbio Biotechnology, Beijing, China). Liver tissues were fixed in 10% neutral formalin solution, embedded in paraffin, and serially sectioned (5 µm thick). The sections were fixed on slides, dewaxed in xylene, and rehydrated in an ethanol gradient. Afterward, the sections were washed with distilled water for 2 min, stained with hematoxylin for 10 min, differentiated for 3 min, and stained with eosin for 2 min. The sections were sealed with glycerol gelatin. Finally, the sections observed and photographed under a digital section scanner (3D HISTECH, DANJIER Electronic Co., Ltd., Jinan, China).

### Cell culture and treatment

HepG2 cells were purchased from the Cell Bank of the Chinese Academy of Sciences (Shanghai, China). The cells were cultured in Dulbecco’s modified Eagle medium (Sparkjade, Shandong, China) containing 10% fetal bovine serum (Thermo Fisher Scientific, Waltham, MA, United States) and 1% penicillin-streptomycin (Thermo Fisher Scientific, Waltham, MA, United States) after which they were passaged or seeded when confluence reached 85%–90%. High-fat HepG2 cell models were induced with 1 mM of free fatty acids (FFAs, Sigma, St. Louis, MO, United States) and treated with different concentrations of SYTZD drug containing serum.

### Cell viability assay

Cell viability was determined using a cell counting kit-8 (CCK-8, Sparkjade, Shandong, China). HepG2 cells were seeded into 96-well plates at a density of 5×10^3^, adhered overnight, and treated with different concentrations of SYTZD drug containing serum for 24 h. Afterwards, 10 µl of CCK-8 solution was added to each well of the 96-well plate, and the cells were incubated at 37°C for 2–3 h. Finally, the absorbance value of each well was measured at 450 nm using a microplate reader (Thermo Fisher Scientific, Waltham, MA, United States).

### RNA extraction and real-time quantitative polymerase chain reaction

Total RNA was extracted from HepG2 cells using RNA extraction kit (Sparkjade, Shandong, China), and RNA purity was detected using Nanodrop 2000c (Thermo Fisher Scientific, Waltham, MA, United States). Total RNA was reverse transcribed to cDNA using reverse transcription kit (Sparkjade, Shandong, China). Quantitative polymerase chain reactions (qPCR) were performed with SYBR Green I Kit (Sparkjade, Shandong, China) in the RT-qPCR Detection System LC480 (Roche, Mannheim, Germany) for a total of 40 cycles (94°C for 20 s, 60°C for 20 s, 72°C for 30 s). Glyceraldehyde-3-phosphate dehydrogenase (GAPDH) was used as an internal reference gene, and mRNA expression levels were calculated using the 2^−ΔΔCT^ method. The sequences of specific primers are listed in Table 1.

**TABLE 1:** Sequences of primers.

Gene	Forward sequence (5′ to 3′)	Reverse sequence (5′ to 3′)
mTOR	GGA​GGC​TGA​TGG​ACA​CAA​AT	CTG​TGG​TCC​CCG​TTT​TCT​TA
FASN	AAG​GAC​CTG​TCT​AGG​TTT​GAT​GC	TGG​CTT​CAT​AGG​TGA​CTT​CCA
ESR1	CCT​CCT​CAT​CCT​CTC​CCA​CAT​CAG	GCA​TCT​CCA​GCA​GCA​GGT​CAT​AG
HIF-1α	CCA​CTG​CCA​CCA​CTG​ATG​AA	TTGGTGA GGCTGTCCGACTT
VEGFA	ATC​GAG​TAC​ATC​TTC​AAG​CCA​T	GTG​AGG​TTT​GAT​CCG​CAT​AAT​C
GSK-3β	GTG​GCA​GAC​AAA​GAA​ATG​TG	AAC​TGA​GAG​CAA​AAC​AAA​ACC
GAPDH	CAG​GGC​TGC​TTT​TAA​CTC​TGG​T	GAT​TTT​GGA​GGG​ATC​TCG​CT

### Protein extraction and Western blotting

Total protein was extracted from HepG2 cells and rat liver using RIPA lysate buffer (Sparkjade, Shandong, China) containing protease inhibitors and phosphatase inhibitors (Beyotime Biotechnology, Shanghai, China), respectively. Protein concentration was determined using BCA kit (Sparkjade, Shandong, China). Twenty micrograms of protein per lane were separated on 7.5% or 10% sodium dodecyl sulfate polyacrylamide gel electrophoresis (SDS-PAGE) gels (Sparkjade, Shandong, China) and electro-transferred to polyvinylidene difluoride membranes after which the membranes were sealed with 5% skimmed milk (Sparkjade, Shandong, China) at room temperature for 1 h. Afterward, the membranes were incubated with several primary antibodies: (Chalasani et al., 2018) estrogen receptor 1 (ESR1, 1:1000, Abcam, Cambridge, United States); (Loomba et al., 2021); mammalian target of rapamycin (mTOR, 1:1000, Bioss, Beijing, China); (Polyzos et al., 2019); phospho-mTOR (p-mTOR, 1:1000, Bioss, Beijing, China); (Wang and Malhi, 2018); fatty acid synthase (FASN, 1:5000, Abcam, Cambridge, United States); (Cotter and Rinella, 2020); hypoxia-inducible factor-1α (HIF-1α, 1:1000, Abcam, Cambridge, United States); (Zhou et al., 2019); vascular endothelial growth factor-A (VEGFA, 1:1000, Abcam, Cambridge, United States); (Kanwal et al., 2021); glycogen synthase kinase-3 beta (GSK-3β, 1:500, Santa Cruz Biotechnology, Shanghai, China); (Bessone et al., 2019); phospho-GSK-3β (p-GSK-3β, 1:500, Santa Cruz Biotechnology, Shanghai, China); and (Fang et al., 2018) β-actin (1:5000, Proteintech, Hubei, China) overnight at 4°C followed by incubation with horseradish peroxidase (HRP)-labeled goat anti-mouse or -rabbit immunoglobulin secondary antibody (1:5000, sparkjade, Shandong, China) at room temperature for 1 h. Finally, ECL chemiluminescence reagent (Sparkjade, Shandong, China) was used to develop the different protein bands. The protein expression levels were analyzed by ImageJ software and normalized to β-actin.

### Statistical analysis

All experiments were repeated for three independent trials. GraphPad Prism 9.0 software was applied for statistical analysis, and results were expressed as mean ± standard deviation. The differences between two groups were examined by multiple t-tests. Multiple groups of independent data were compared using one-way analysis of variance (ANOVA). Values of *p* < 0.05 were considered statistically significant.

## Results

### Identification of chemical components of SYTZD and constituents in rat serum

**FIGURE 2:**
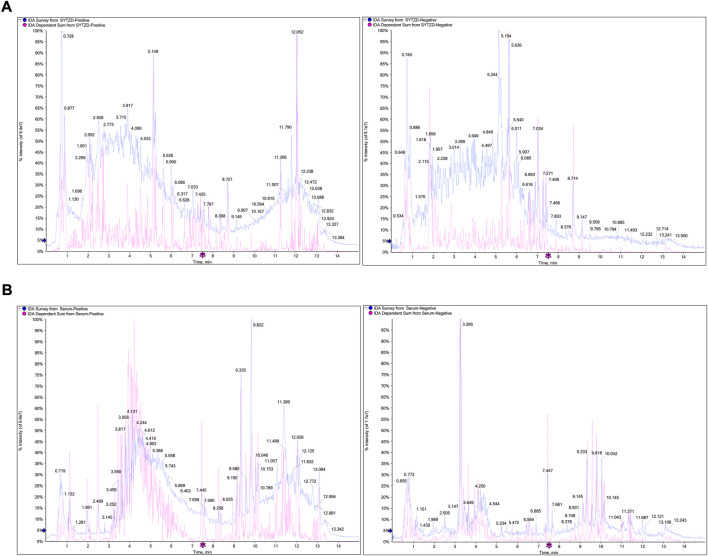
UPLC-Q/TOF-MS analysis of chemical compounds in SYTZD and rat serum. **(A)** Total ion current (TIC) chromatogram of SYTZD under positive ion mode and negative ion mode. **(B)** Total ion current (TIC) chromatogram in rat serum under the positive ion mode and negative ion mode.

The chemical components of SYTZD were analyzed with UPLC-Q/TOF-MS and the results were presented in Figure 2A and Supplementary Table S1. The collected mass spectrometry data was analyzed by SCIEX OS software. Based on the first-order accurate mass number, isotope distribution ratio and MS/MS data of the compounds and the TCM MS/MS library in SCIEX OS software, 84 compounds were identified under positive ion mode, and 119 compounds were identified under the negative ion mode from SYTZD. Analytical results for constituents in rat serum were shown in Figure 2B and Supplementary Table S2. 60 compounds were identified under positive ion mode, and 74 compounds were identified under the negative ion mode from SYTZD. In addition, two constituents identified in rat serum were coincident with the compounds identified from SYTZD, including diosgenin and stigmasterol. The results indicated that SYTZD might exert functions through diosgenin and stigmasterol.

### Screening of active constituents and potential therapeutic targets of SYTZD

**FIGURE 3:**
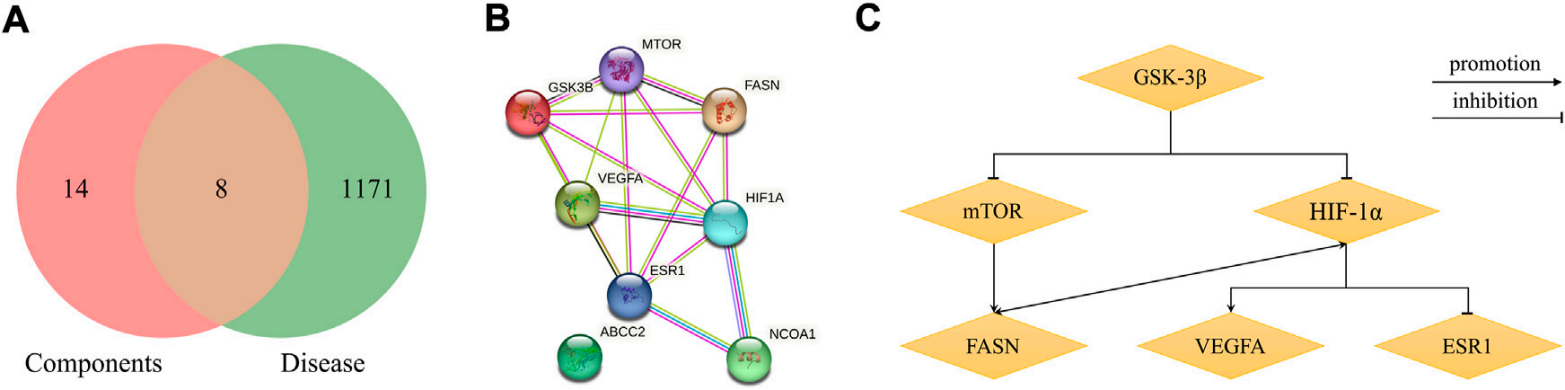
Network analysis of common targets of diosgenin and stigmasterol against NAFLD. **(A)** Common targets in Venn diagram. **(B)** A PPI network diagram of common targets. **(C)** Clustering analysis diagram of common targets.

According to the criteria of OB ≥ 30% and DL ≥ 0.18 in the TCMSP database, 17 bioactive components were screened (Table 2). A total of 32 corresponding component targets were obtained from the TCMSP database, and then verified in the ETCM database. Among these targets, 20 targets of diosgenin and stigmasterol were identified. Another two targets of diosgenin and stigmasterol, namely, GSK-3β and ESR1, were also included for further analysis according to previous studies (Jayaraman et al., 2020; Wang et al., 2021a). Besides, 1179 NAFLD-related targets were collected from the GeneCards database, and then verified in the DisGeNET database. Finally, 8 common targets were identified by mapping 1179 disease targets and 22 component targets of diosgenin and stigmasterol using the Venny platform (Figure 3A).

**TABLE 2:** Major components of SYTZD by network pharmacology analysis.

Molecule name	OB (%)	DL	Compound structure	CAS
diosgenin	80.88	0.81	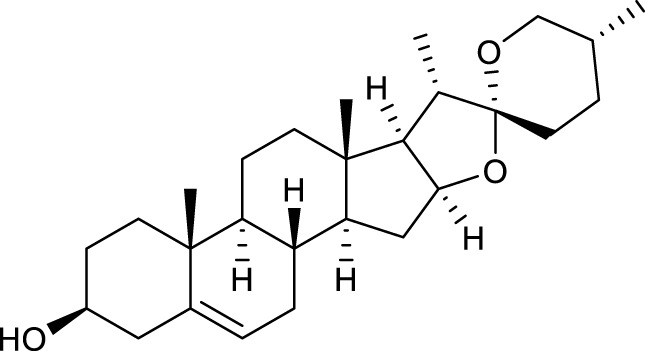	512-04-9
hancinol	64.01	0.37	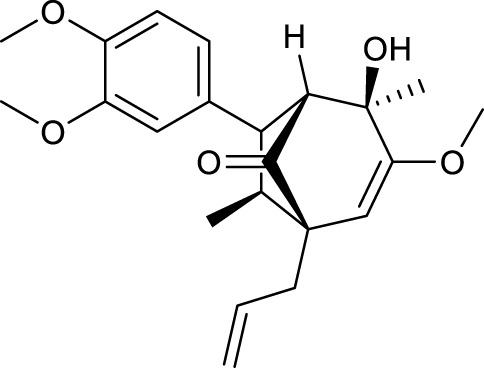	108864-50-2
Denudatin B	61.47	0.38	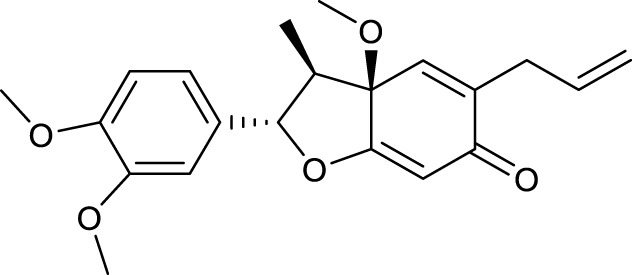	87402-88-8
(-)-taxifolin	60.51	0.27	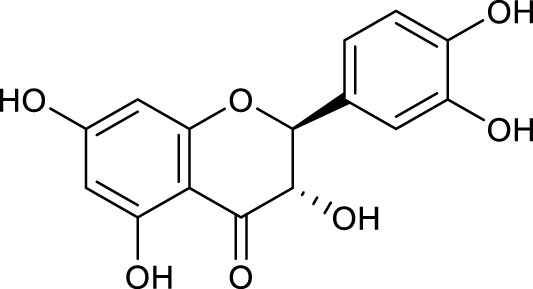	480-18-2
hancinone C	59.05	0.39	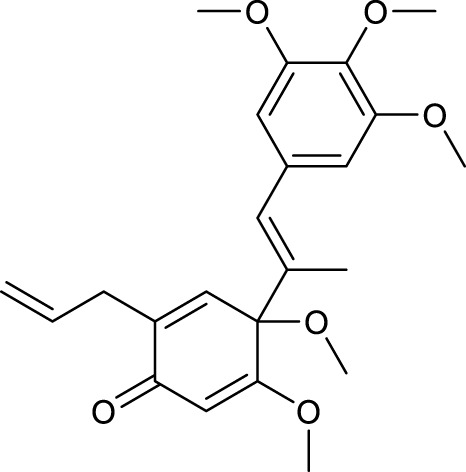	111843-10-8
Kadsurenone	54.72	0.38	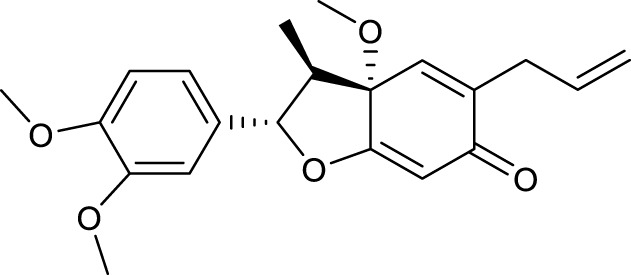	95851-37-9
AIDS180907	45.33	0.77	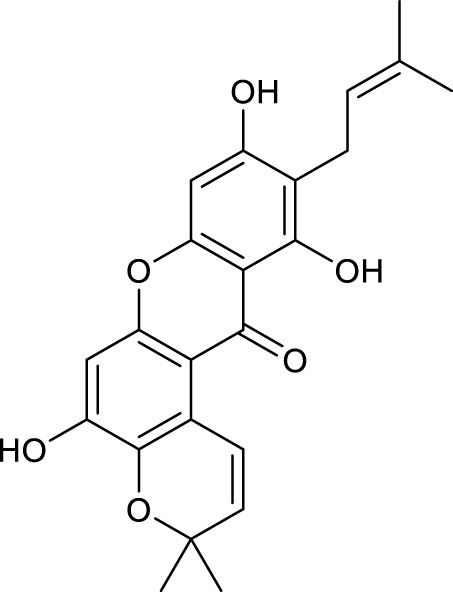	76996-28-6
Stigmasterol	43.83	0.76	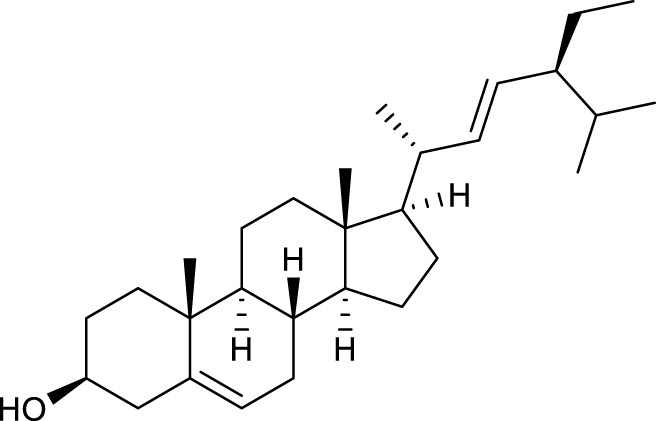	83-48-7
Isofucosterol	43.78	0.76	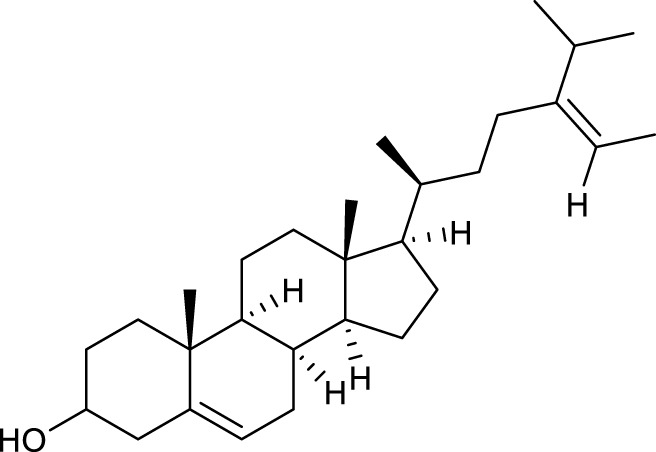	18472-36-1
Doradexanthin	38.16	0.54	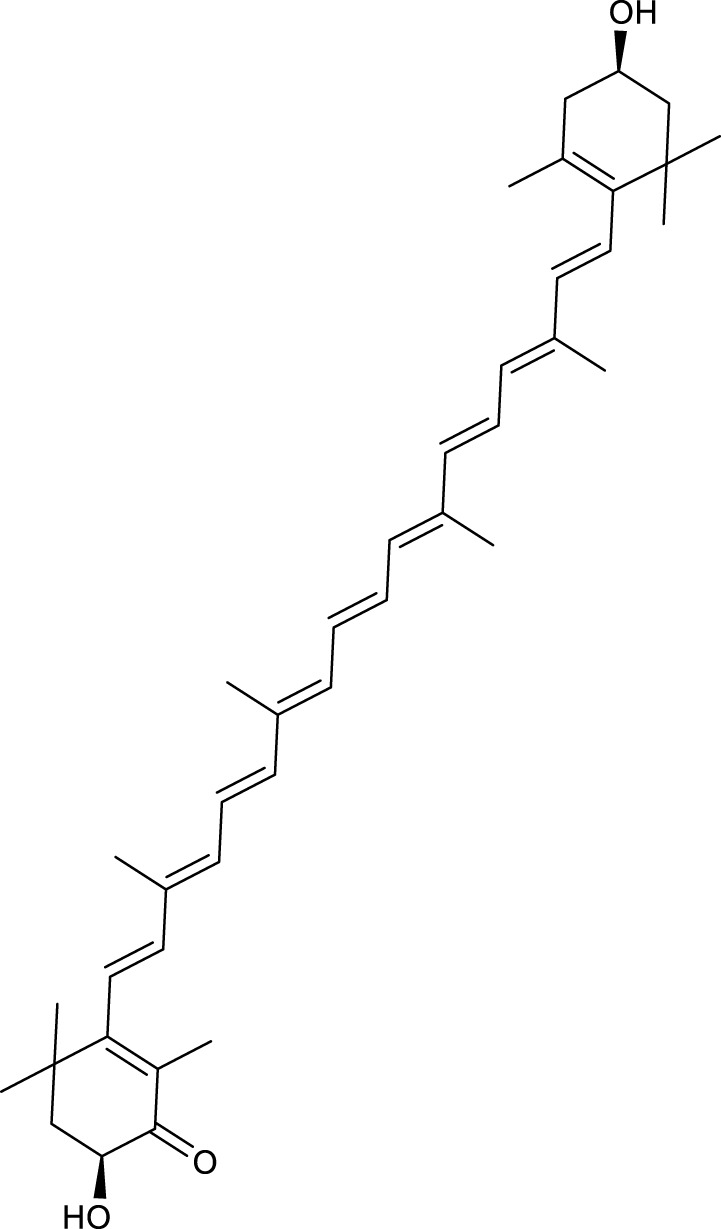	4418-73-9
CLR	37.87	0.68	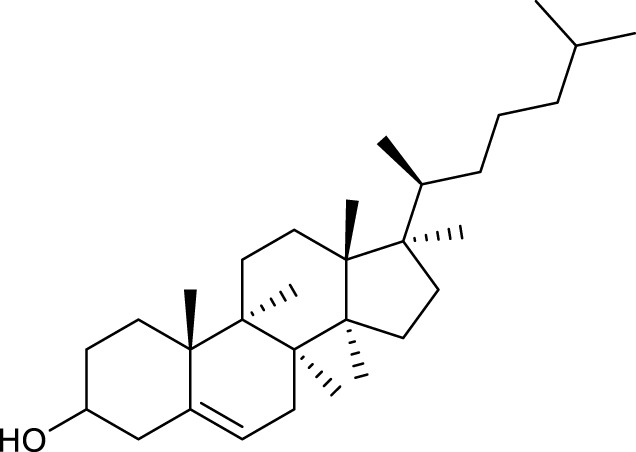	57-88-5
24-Methylcholest-5-enyl-3belta-O-glucopyranoside_qt	37.58	0.72	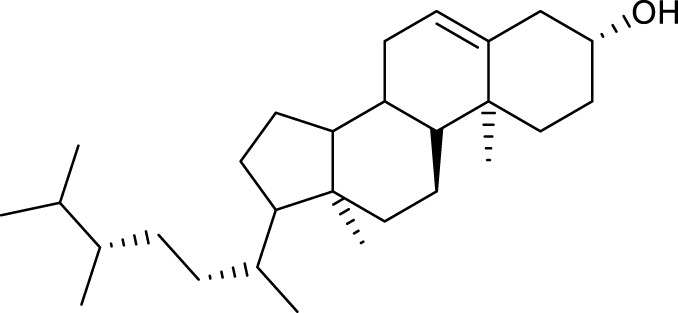	474-63-5
Campesterol	37.58	0.71	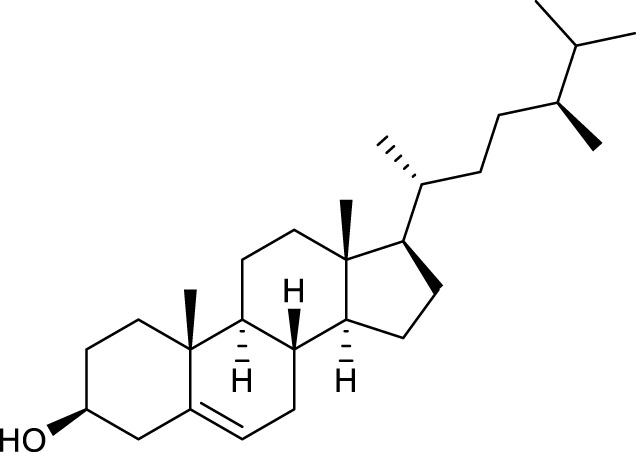	474-62-4
Dioscoreside C_qt	36.38	0.87	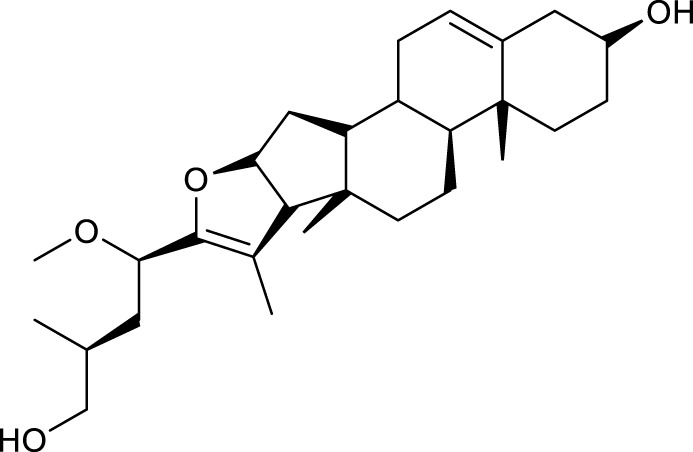	344912-80-7
Methylcimicifugoside_qt	31.69	0.24	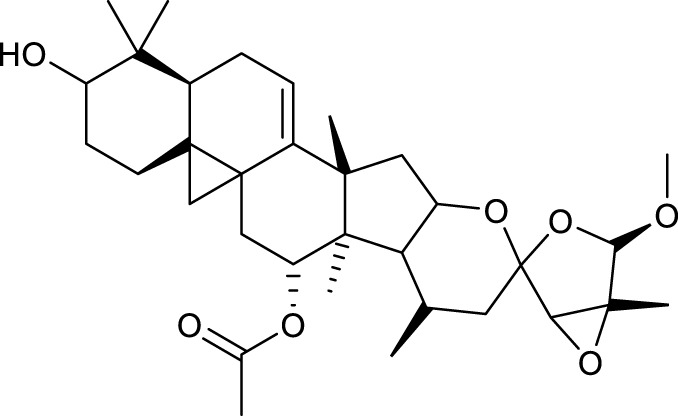	66176-93-0
piperlonguminine	30.71	0.18	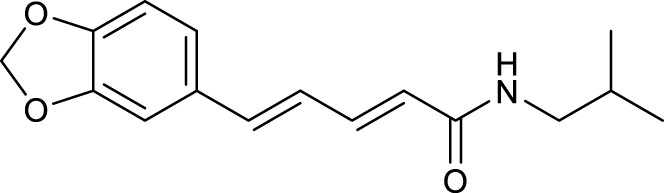	5950-12-9
EINECS 213-897-0	71.96	0.72	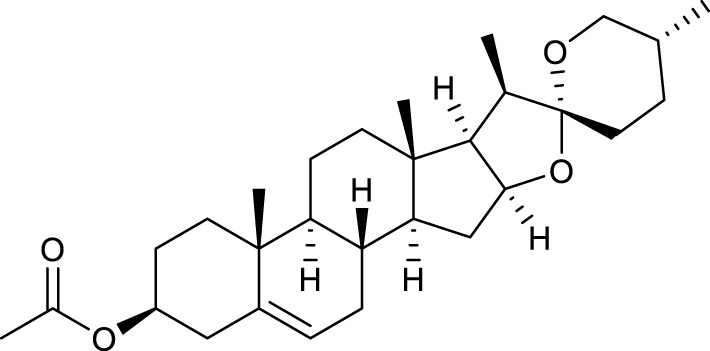	1061-54-7

Note: OB, oral bioavailability; DL, drug likeness.

### PPI network construction and core target selection

The 8 common targets were imported into the STRING platform, and a PPI network was generated, which included 8 points and 16 edges (Figure 3B). A cluster analysis of the PPI network was performed by MCODE plugin in Cytoscape software. One cluster and six core genes were obtained, and the core genes included ESR1, FASN, mTOR, HIF-1α, VEGFA, and GSK-3β (Figure 3C). These results indicated that SYTZD treated NAFLD probably *via* modulation of ESR1, FASN, mTOR, HIF-1α, VEGFA, and GSK-3β.

### KEGG pathway enrichment analysis

**FIGURE 4:**
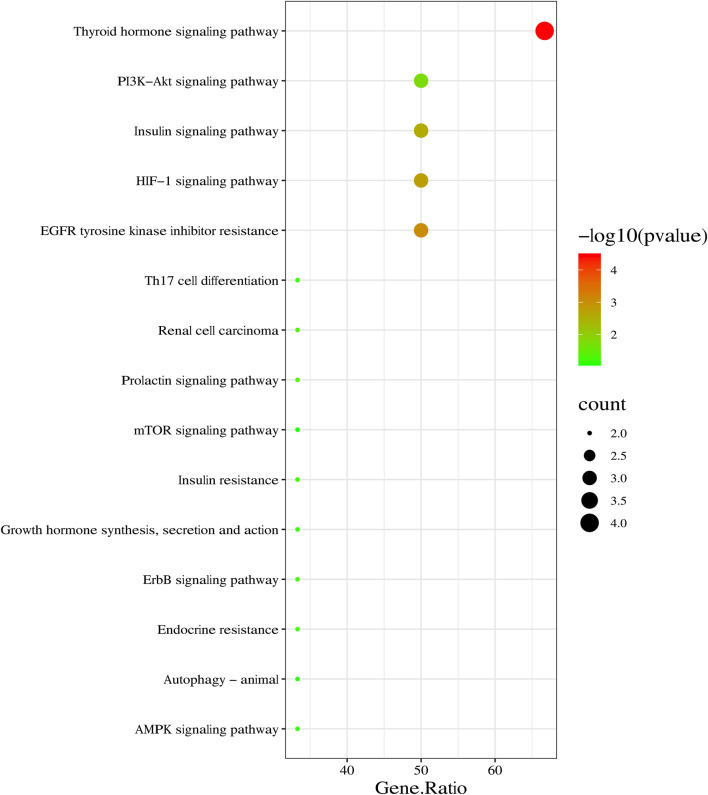
KEGG pathway enrichment analysis of core targets of SYTZD against NAFLD. Top 15 KEGG pathways are listed in the bubble chart.

The core targets (ESR1, FASN, mTOR, HIF-1α, VEGFA, and GSK-3β) of SYTZD against NAFLD were imported into the DAVID database for KEGG analysis. The results showed that 31 signaling pathways of the core targets were obtained. The top 15 pathways were identified based on the number of enriched genes and *p*-values (Figure 4). Among these pathways, thyroid hormone (ZHU et al., 2020), insulin resistance (Tanase et al., 2020), HIF-1 (Mesarwi et al., 2021), mTOR, and AMPK signaling pathways (Park et al., 2021) were found to be closely associated with NAFLD.

### SYTZD reduced body weight and liver weight ratio in NAFLD rats

**FIGURE 5:**
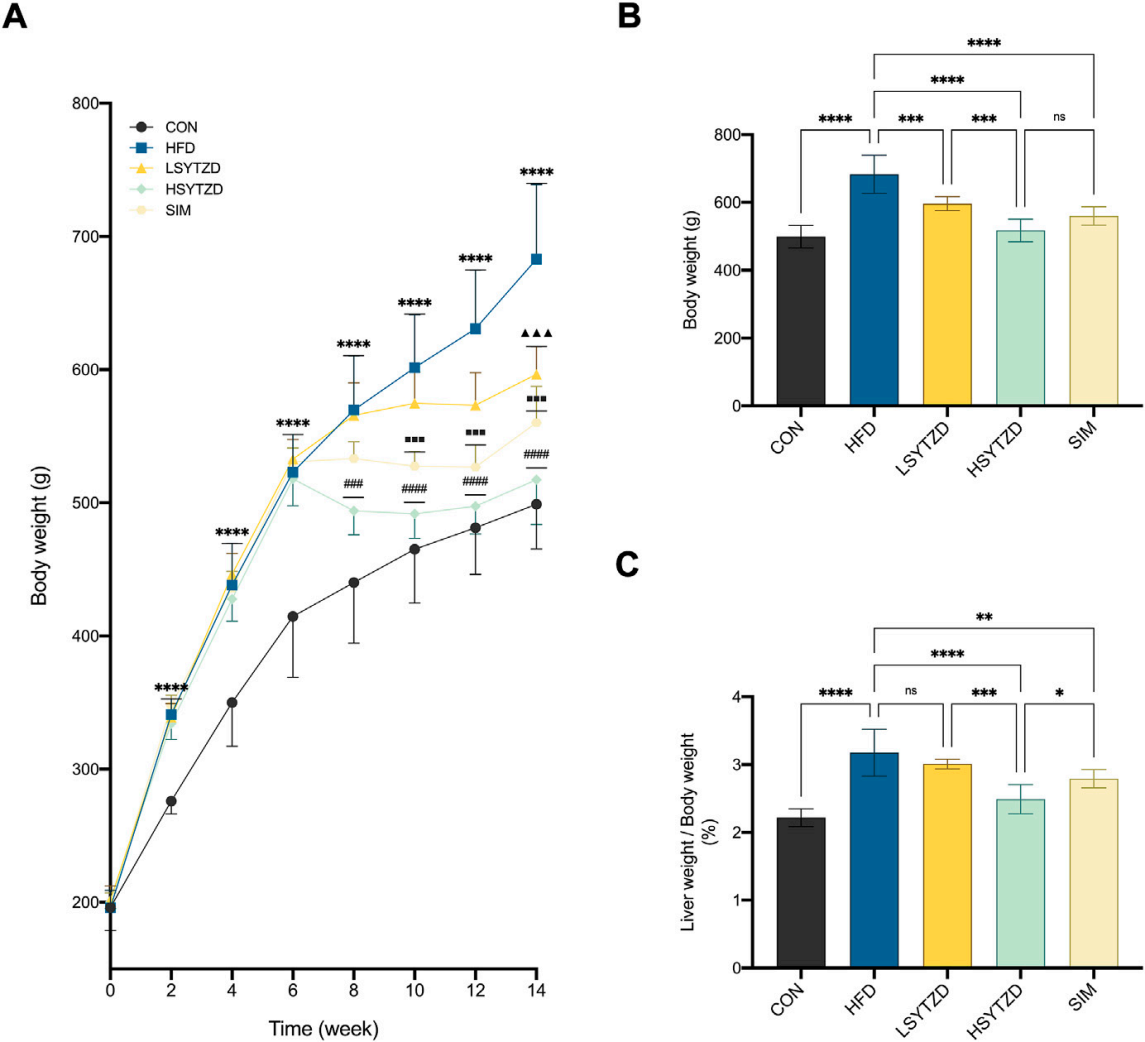
Effect of SYTZD on body weight and liver weight ratio in NAFLD rats. **(A)** Comparison of body weight gain in rats over the 14-week study (*n* = 8). **(B)** Comparison of body weight in rats after 8-week drug intervention (*n* = 8). **(C)** Ratio of liver weight/body weight in rats after 8-week drug intervention (*n* = 8). In **(A)** ****p < 0.0001 *versus* CON group; ^▲▲▲^ *p* < 0.001 *versus* HFD group; ^###^ *p* < 0.001, ^####^ *p* < 0.0001 *versus* HFD group; ^■■■^ *p* < 0.001 *versus* HFD group. In **(B,C)** *p < 0.05, **p < 0.01, ***p < 0.001, ****p < 0.0001.

During the 14-week research period, the body weight gains of all rats increased concurrently before treatment (Figure 5A). The body weight gains were significantly higher in rats fed HFD than that of the rats fed normal diets (Figure 5A). Compared with the HFD group, the body weight gains of rats in the LSYTZD, HSYTZD, and SIM groups slowed during long-term administration (Figure 5A). After the 8-week drug intervention, the body weights of rats obviously increased in the HFD group when compared with the CON group (Figure 5B). The body weights significantly decreased in the LSYTZD and HSYTZD groups when compared with the HFD group (Figure 5B). SYTZD caused these changes in a dose-dependent manner, and the inhibitory effect of HSYTZD on the increase in body weights of HFD-fed rats was similar to that in the SIM group (Figure 5B). Consistently, the liver weight ratios of rats were significantly higher in the HFD group than that of the CON group (Figure 5C). The liver weight ratios of rats were markedly lower in the HSYTZD group than that of the HFD group (Figure 5C). The inhibitory effect of HSYTZD on the increase in liver weight ratios of HFD-fed rats was more noticeable than that in the SIM group (Figure 5C). These results indicate that SYTZD could lead to a reduction in HFD-induced body weight and liver weight ratio in NAFLD rats.

### SYTZD improved blood lipid disorder and liver lipid deposition in NAFLD rats

**FIGURE 6:**
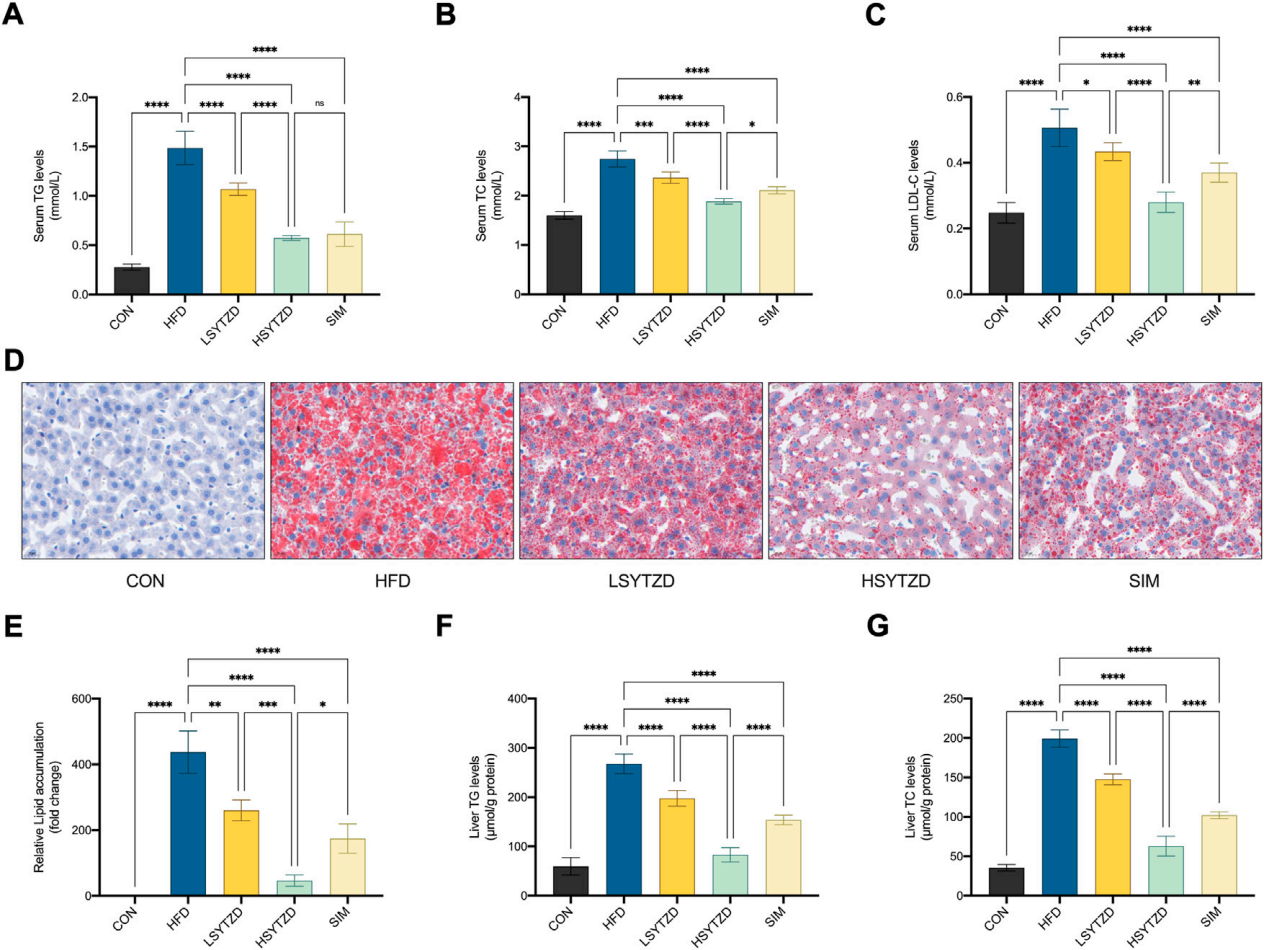
Effect of SYTZD on TC, TG, and LDL-C levels and liver lipid deposition in NAFLD rats. **(A–C)** The levels of serum TC, TG and LDL-C after the 8-week drug intervention. (*n* = 5). **(D)** Liver lipid deposition stained with Oil Red O staining (400 ×) after 8-week drug intervention. **(E)** Area of Oil Red O staining was quantified by ImageJ software. **(F,G)** The levels of liver TC and TG after 8-week drug intervention. *p < 0.05, **p < 0.01, ***p < 0.001, ****p < 0.0001.

The results showed that the levels of serum TG, TC, and LDL-C significantly increased in the HFD group when compared with the CON group (Figures 6A–C). SYTZD treatment caused a significant reduction in the levels, and SYTZD induced these changes in a dose-dependent way (Figures 6A–C). The inhibitory effect of HSYTZD on the increase in TC and LDL-C levels of HFD-fed rats was more noticeable than in the SIM group (Figures 6B,C). These results indicate that SYTZD could lead to an improvement in HFD-induced dyslipidemia in NAFLD rats.

Oil red O staining in liver tissue showed that the lipid deposition caused a significant increase in the HFD group when compared with the CON group (Figures 6D,E). SYTZD administration led to a marked reduction in lipid deposition, and SYTZD caused these changes in a dose-dependent way (Figures 6D,E). The inhibitory effect of HSYTZD on the lipid deposition of HFD-fed rats was better than that of SIM group (Figures 6D,E). Consistently, the levels of liver TG and TC were significantly higher in the HFD group than those in the CON group (Figures 6F,G). The levels of liver TG and TC were significantly lower in the LSYTZD and HSYTZD groups than those in the HFD group (Figures 6F,G). The HSYTZD-induced decreases in the levels of liver TG and TC were more noticeable than those in the SIM group (Figures 6F,G). These results indicate that SYTZD could lead to an improvement in HFD-induced liver lipid deposition in NAFLD rats.

### SYTZD ameliorated hepatic steatosis and liver injury in NAFLD rats

**FIGURE 7:**
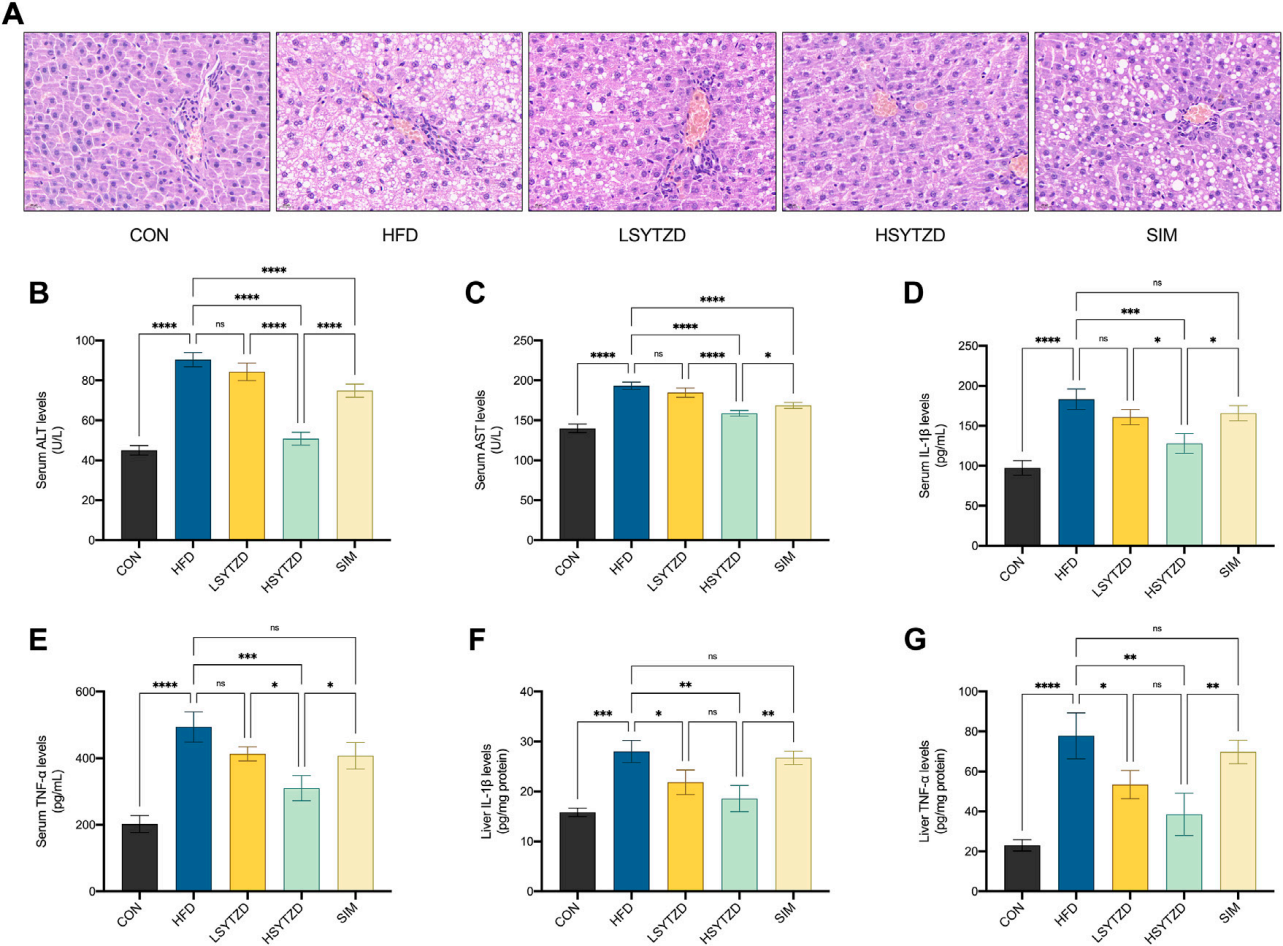
Effect of SYTZD on liver histopathology and ALT, AST, IL-1β, and TNF-α levels in NAFLD rats. **(A)** Liver histopathology stained with H&E staining (400 ×) after the 8-week drug intervention. **(B,C)** The levels of serum ALT and AST after the 8-week drug intervention (*n* = 3). **(D–G)** The levels of IL-1β, and TNF-α in the serum and liver after the 8-week drug intervention (*n* = 3). *p < 0.05, **p < 0.01, ***p < 0.001, ****p < 0.0001.

H&E staining showed that the hepatocyte cords were disordered, hepatocytes were swollen, and obvious large vesicular steatosis could be seen in the HFD group (Figure 7A). The liver tissue was normal in structure with neatly arranged hepatocyte cords and well-defined liver lobules, and no hepatic steatosis was observed in the CON group (Figure 7A). When compared with the HFD group, SYTZD intervention caused a marked reduction in the degree of hepatocyte steatosis, alleviation in cytoplasmic vacuolization-like changes, and a reduction or disappearance of lipid droplets (Figure 7A). SYTZD caused the changes in a dose-dependent manner, and the inhibitory effect of HSYTZD on the hepatocyte steatosis of HFD-fed rats was more noticeable than in the SIM group (Figure 7A). These results indicate that SYTZD could lead to an improvement in HFD-induced hepatocyte steatosis in NAFLD rats.

To study HFD-induced hepatocyte damage, we measured the levels of serum ALT and AST, which are the most sensitive indicators of hepatocyte damage (Wang et al., 2021b). The results showed that the levels of serum ALT and AST had significantly increased in the HFD group when compared with the CON group (Figures 7B,C). The levels of serum ALT and AST significantly decreased in the HSYZTD group when compared with the HFD group (Figures 7B,C). The HSYTZD-induced decrease in the levels of serum ALT and AST was more noticeable than that in the SIM group (Figures 7B,C). In addition, we explored whether excessive lipid accumulation in liver contributed to inflammation. The results from serum samples showed that the levels of serum IL-1β and TNF-α were significantly higher in the HFD group than that of the CON group (Figures 7D,E). The levels of serum IL-1β and TNF-α were significantly lower in the HSYTZD group than that in the HFD group (Figures 7D,E). The HSYTZD-induced decrease in the levels of serum IL-1β and TNF-α was more noticeable than that in the SIM group (Figures 7D,E). Results from the liver samples showed that the levels of liver IL-1β and TNF-α were markedly higher in the HFD group those that in the CON group (Figures 7F,G). SYTZD treatment could lead to a reduction in the levels of liver IL-1β and TNF-α, and SYTZD caused these changes in a dose-dependent manner (Figures 7F,G). The HSYTZD-induced decrease in the levels of liver IL-1β and TNF-α was more noticeable than that in the SIM group (Figures 7F,G). These results indicate that HFD induced liver damage and led to an increase in inflammatory factors and serum transaminase levels in NAFLD rats. SYTZD treatment ameliorated the liver damage caused by large amount of fat intake in NAFLD rats.

### SYTZD improved insulin sensitivity and alleviated insulin resistance in NAFLD rats

**FIGURE 8:**
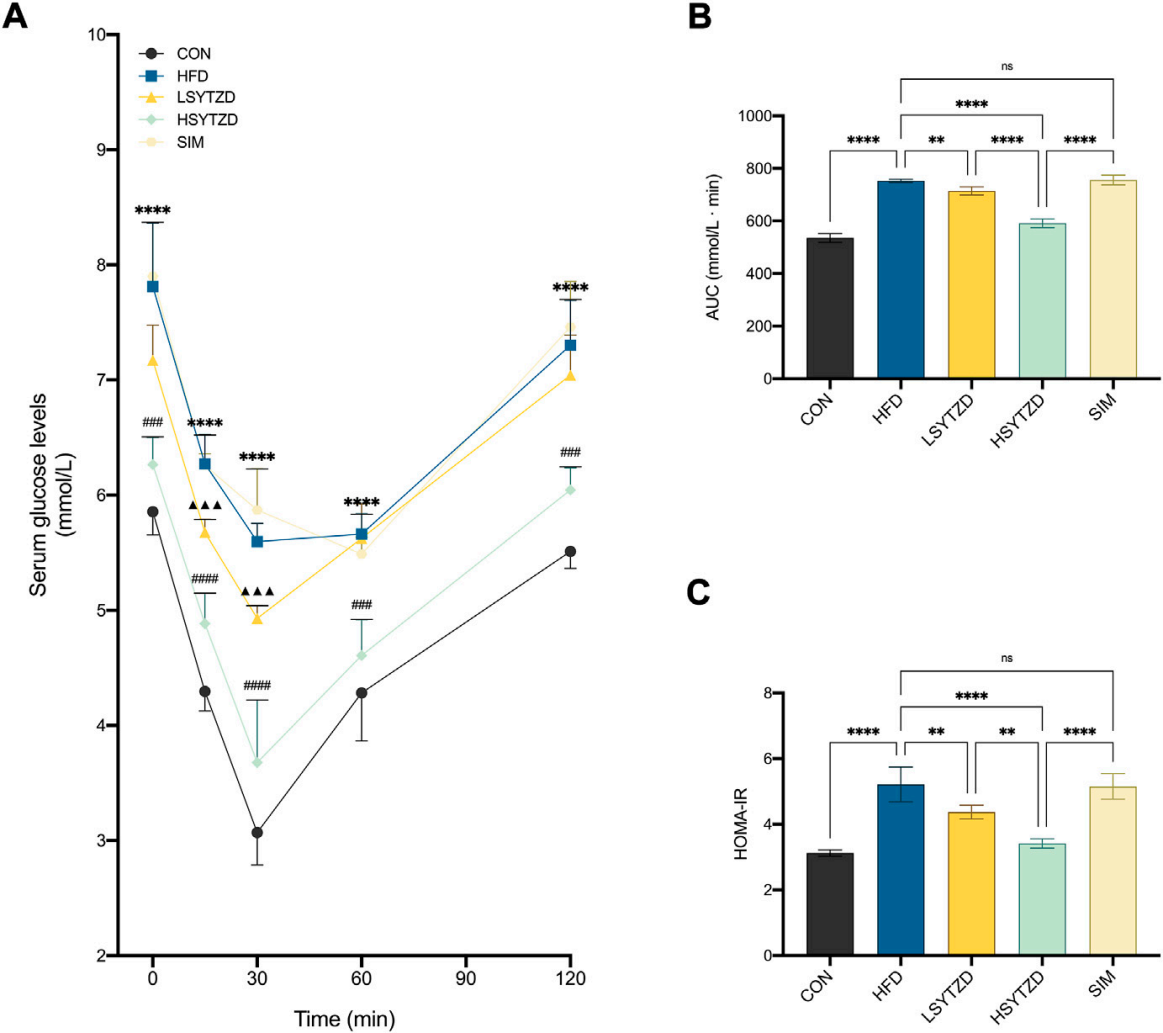
Effect of SYTZD on blood glucose and insulin in NAFLD rats. **(A)** Changes of blood glucose in ITT after the 8-week drug intervention (*n* = 5). **(B)** AUC of blood glucose in ITT. **(C)** Degrees of HOMA-IR after the 8-week drug intervention (*n* = 5). In **(A)** ****p < 0.0001 *versus* CON group; ^▲▲▲^ *p* < 0.001 *versus* HFD group; ^###^ *p* < 0.001, ^####^ *p* < 0.0001 *versus* HFD group. In **(B,C)** **p < 0.01, ****p < 0.0001.

ITT results showed that blood glucose levels were consistently higher in the HFD group than those in the CON group at 0–120 min (Figure 8A). The blood glucose level decreased in the LSYTZD group when compared with the HFD group at 15 min and 30 min but not at 60–120 min (Figure 8A). The blood glucose decreased in the HSYTZD group when compared with the HFD group at 0–120 min (Figure 8A). However, no difference in blood glucose between the SIM and HFD groups was detected (Figure 8A). The quantitative data of the ITT curve showed that the AUC with SYTZD intervention was significantly lower than that of the HFD group, and SYTZD caused these changes in a dose-dependent way (Figure 8B). Additionally, HOMA-IR was significantly higher in the HFD group than in the CON group. HOMA-IR with SYTZD intervention was significantly lower than that in the HFD group, and SYTZD caused the changes in a dose-dependent way (Figure 8C). These results indicate that SYTZD led to an improvement in HFD-induced insulin sensitivity and alleviated insulin resistance in NAFLD rats. The HSYTZD-induced improvement in the insulin resistance of HFD-fed rats was more noticeable than that in the SIM group.

### SYTZD affected relative protein levels of mTOR, FASN, ESR1, HIF-1α, VEGFA, and GSK-3β in NAFLD rats

**FIGURE 9:**
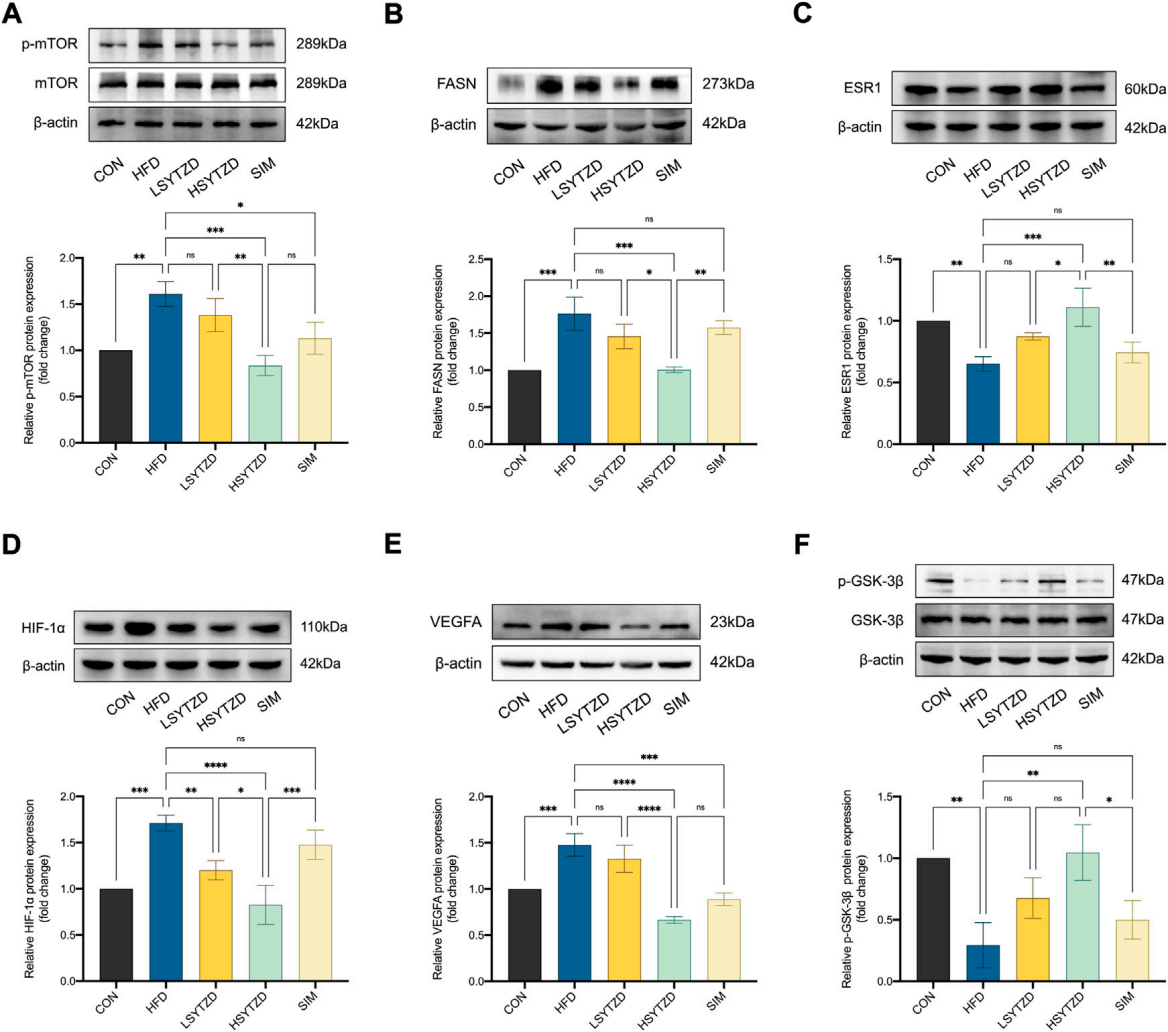
Effect of SYTZD on relative protein levels of ESR1, mTOR, FASN, HIF-1α, VEGFA, and GSK-3β in NAFLD rats. **(A–F)** Relative protein levels of mTOR, FASN, ESR1, HIF-1α, VEGFA, and GSK-3β in liver of NAFLD rats. *p < 0.05, **p < 0.01, ***p < 0.001, ****p < 0.0001.

To investigate the underlying mechanisms of SYTZD against NAFLD, we performed experimental validation of the relevant pathway proteins predicted by network pharmacology. WB analysis showed that relative protein levels of p-mTOR and FASN had obviously increased in the HFD group when compared with the CON group (Figures 9A,B). Relative protein levels of p-mTOR and FASN decreased in the LSYTZD and HSYTZD groups when compared with the HFD group, and SYTZD caused these changes in a dose-dependent way (Figures 9A,B). The HSYTZD-induced decrease in the relative protein level of FASN was more noticeable than that in the SIM group (Figure 9B). Relative protein level of ESR1 significantly decreased in the HFD group compared with the CON group (Figure 9C). Relative protein levels of ESR1 significantly increased in the HSYTZD group when compared with the HFD group (Figure 9C). The HSYTZD-induced increase in the level of ESR1 was more noticeable than in the SIM group (Figure 9C). Relative protein levels of HIF-1α and VEGFA significantly increased in the HFD group when compared with the CON group (Figures 9D,E). SYTZD treatment led to a decrease in the levels of HIF-1α, and SYTZD and caused these changes in a dose-dependent way (Figure 9D). The HSYTZD-induced decrease in the level of HIF-1α was more noticeable than in the SIM group (Figure 9D). The relative protein level of VEGFA obviously decreased in the HSYTZD group when compared with the HFD group (Figure 9E). The relative protein level of p-GSK-3β markedly decreased in the HFD group when compared with the CON group (Figure 9F). The relative protein level of p-GSK-3β obviously decreased in the HSYTZD group when compared with the HFD group (Figure 9F). The HSYTZD-induced increase in the level of HIF-1α was more noticeable than in the SIM group (Figure 9F). These results indicate that SYTZD treatment led to a reduction in relative protein levels of mTOR, FASN, HIF-1α, and VEGFA and enhancement in relative protein levels of ESR1and p-GSK-3β in livers of NAFLD rats.

### SYTZD drug containing serum increased the viability of HepG2 cells treated with FFAs

**FIGURE 10:**
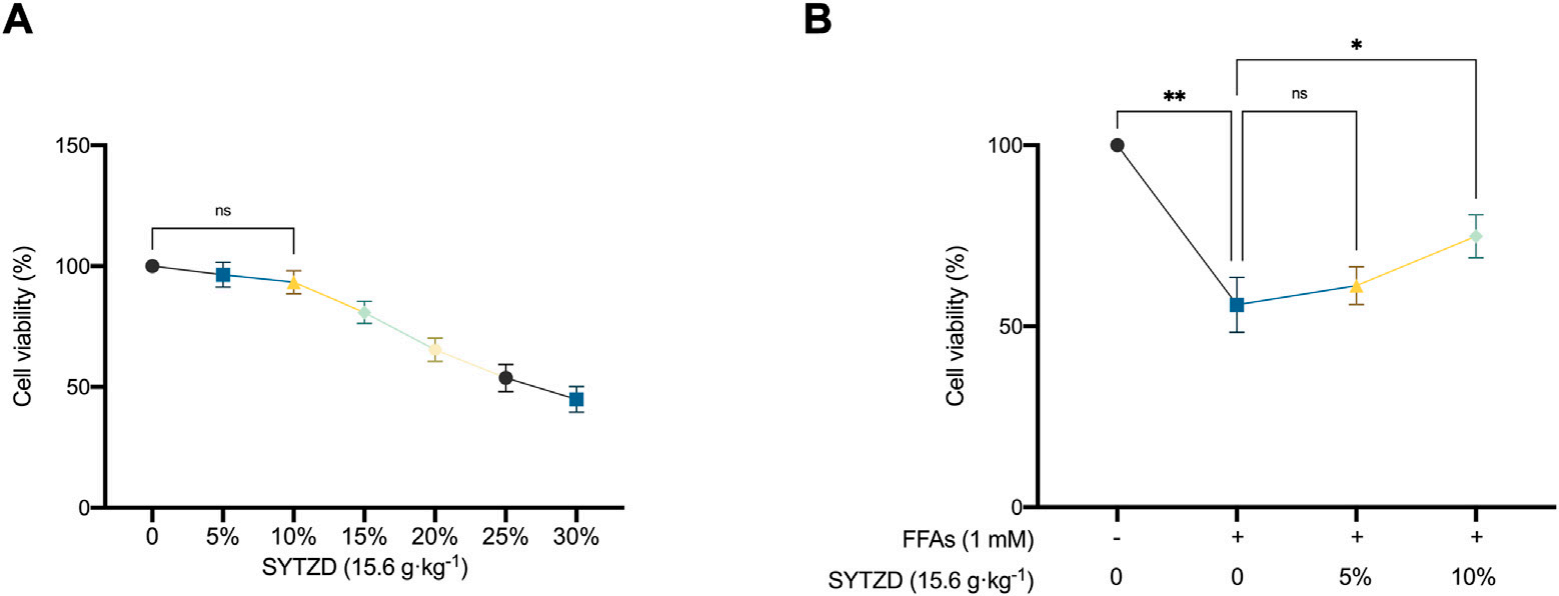
Effects of different concentration of SYTZD drug containing serum on the viability of HepG2 cells. **(A)** Changes of cell viability after intervention with different concentrations of SYTZD drug containing serum. **(B)** Changes of cell viability after intervention with FFAs and SYTZD drug containing serum. *p < 0.05, **p < 0.01.

CCK-8 results showed that SYTZD drug containing serum at concentrations of 0%–10% had no effects on cell activity (Figure 10A), indicating that SYTZD drug containing serum at 0%–10% might be safe concentrations for administration. Furthermore, we determined the effects of different safe concentrations (5%, 10%) of SYTZD drug containing serum on the activity of HepG2 cells induced with 1 mM FFAs. The results showed that the 10% SYTZD drug containing serum caused a significant increase in cell activity, but 5% SYTZD drug containing serum had no effect on this activity (Figure 10B). These results indicate that 10% SYTZD drug containing serum might be a safe and effective concentration for intervention in high-fat cell models.

### SYTZD drug containing serum reduced lipid accumulation and TG, TC contents in HepG2 cells treated with FFAs

**FIGURE 11:**
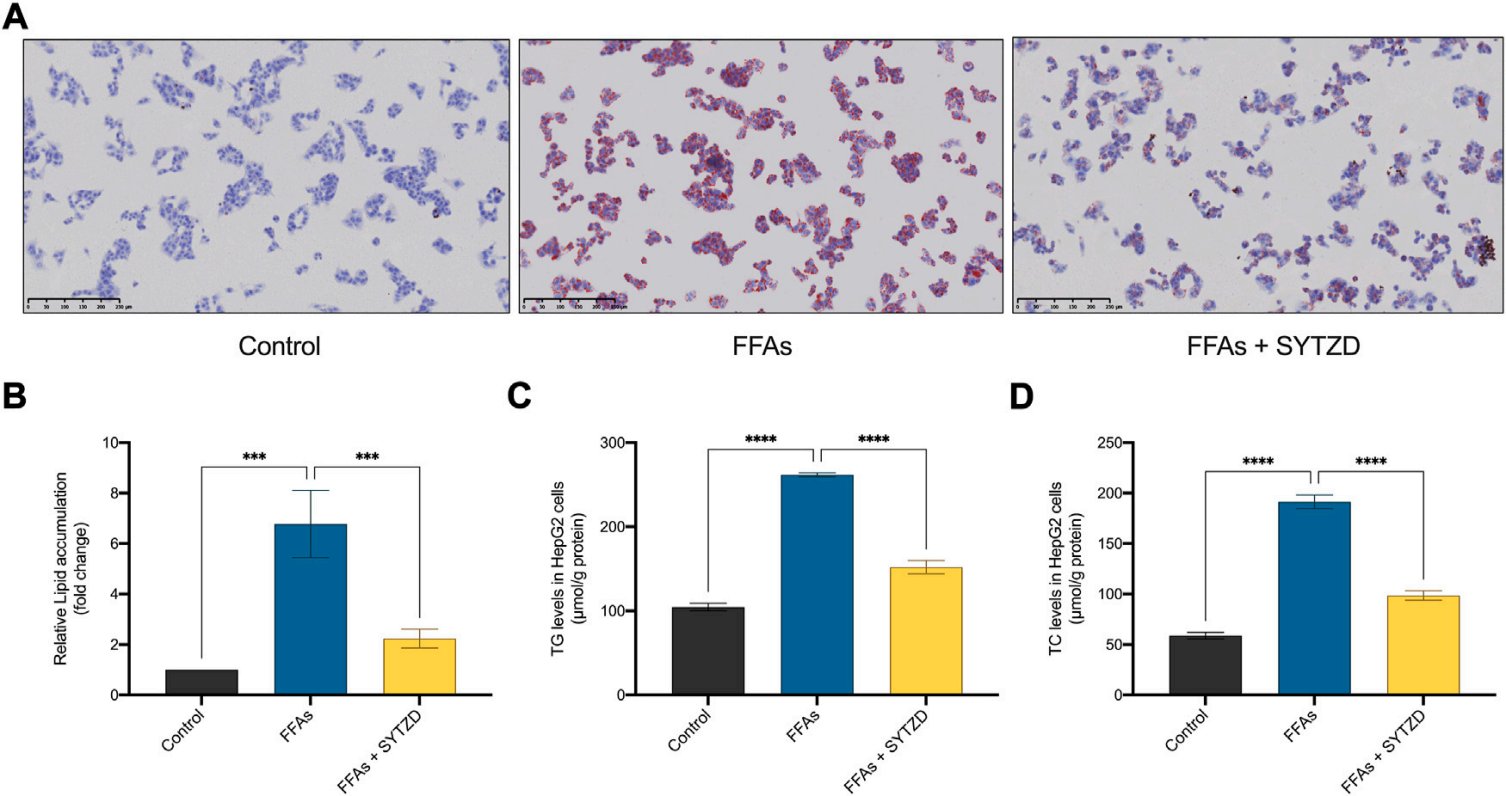
Effect of SYTZD drug containing serum on lipid accumulation in HepG2 cells treated with FFAs. **(A)** Intracellular lipid accumulation stained with Oil Red O staining (200 ×). **(B)** Intracellular lipid accumulation was quantified by ImageJ software. **(C,D)** The levels of TG and TC in HepG2 cells. ***p < 0.001, ****p < 0.0001.

Oil Red O staining in cells showed that FFAs induced significant lipid accumulation in HepG2 cells (Figures 11A,B). SYTZD treatment led to a significant reduction in lipid accumulation (Figures 11A,B). Consistently, intracellular TG and TC levels were significantly higher in the FFA group than in the control group (Figures 11C,D). SYTZD treatment caused a significant reduction in the elevated levels of TG and TC (Figures 11C,D). These results indicate that SYTZD treatment inhibited FFA-induced lipid accumulation in HepG2 cells.

### SYTZD drug containing serum affected relative mRNA and protein levels of mTOR, FASN, ESR1, HIF-1α, VEGFA, and GSK-3β in HepG2 cells treated with FFAs

**FIGURE 12:**
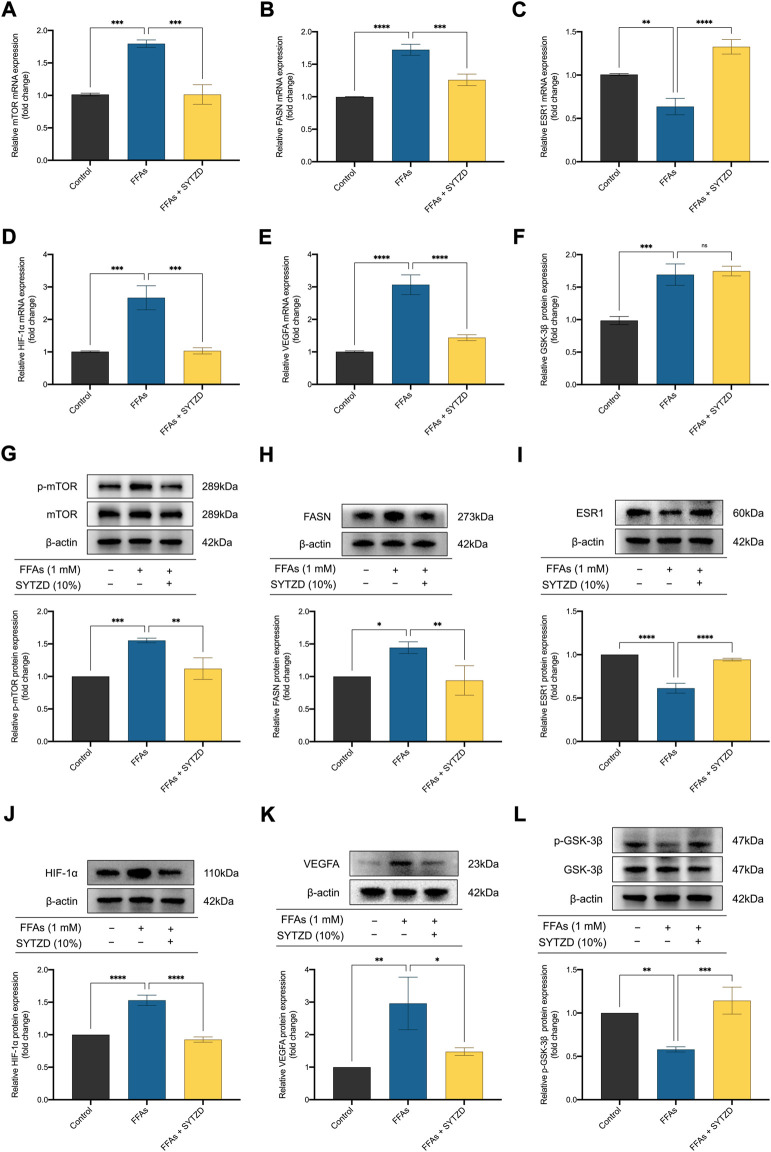
Effect of SYTZD drug containing serum on relative mRNA and protein levels of mTOR, FASN, ESR1, HIF-1α, VEGFA, and GSK-3β in HepG2 cells treated with FFAs. **(A–F)** Relative mRNA levels of mTOR, FASN, ESR1, HIF-1α, VEGFA, and GSK-3β in HepG2 cells. **(G–L)** Relative WB bands and protein levels of mTOR, FASN, ESR1, HIF-1α, VEGFA, and GSK-3β in HepG2 cells. *p < 0.05, **p < 0.01, ***p < 0.001, ****p < 0.0001.

RT-qPCR analysis showed that relative mRNA levels of mTOR and FASN significantly increased in the FFA group when compared with the control group (Figures 12A,B). SYTZD treatment led to an obvious reduction in mTOR and FASN levels (Figures 12A,B). The relative mRNA level of ESR1 significantly decreased in the FFAs group when compared with the control group (Figure 12C). SYTZD treatment caused an obvious increase in the level of ESR1 (Figure 12C). Relative mRNA levels of HIF-1α and VEGFA increased significantly in the FFA group when compared with the control group (Figures 12D,E). SYTZD treatment obviously reduced the levels of HIF-1α and VEGFA (Figures 12D,E). Relative mRNA level of GSK-3β significantly increased in the FFAs group compared with the control group (Figure 12F). However, no difference in GSK-3β mRNA levels between the SYTZD and FFAs groups were found (Figure 12F). WB analysis showed that relative protein levels of p-mTOR and FASN significantly increased in the FFAs group when compared with the control group, while SYTZD intervention led to a significant reduction in the levels of p-mTOR and FASN (Figures 12G,H). The relative protein level of ESR1 significantly decreased in the FFAs group when compared with the control group, while SYTZD intervention caused a significant reduction in level of ESR1 (Figure 12I). The relative protein levels of HIF-1α and VEGFA significantly increased in the FFAs group compared with the control group (Figures 12J,K). SYTZD intervention obviously reduced the levels of HIF-1α and VEGFA (Figures 12J,K). The relative protein level of p-GSK-3β markedly decreased in the FFA group when compared with the control group, whereas SYTZD intervention led to a significant increase in the level of p-GSK-3β (Figure 12L). These results indicate that SYTZD treatment could lead to a reduction in relative mRNA and protein levels of mTOR, FASN, HIF-1α, and VEGFA and an enhancement in the relative mRNA and protein levels of ESR1and p-GSK-3β in HepG2 cells treated with FFAs.

## Discussion

The prevalence of NAFLD is rapidly becoming a serious public health problem, and the resulting incidence from liver cirrhosis, hepatocellular carcinoma, and mortality due to liver-related diseases continues to increase (Huang et al., 2021b). Currently, no specific drugs or standardized treatment protocols for NAFLD are available (Sumida and Yoneda, 2018), and the treatment of NAFLD with TCM is gradually attracting the attention of clinicians (Younossi et al., 2019). TCM formulas are based on the material basis of multiple active components, act on multiple targets for exerting their efficacy, and have unique clinical and scientific values (Huang et al., 2018). Clinical trials have shown that TCM formulas can lead to improvements in NAFLD with low adverse effects and good efficacy (Dang et al., 2020). Our previous studies have found that SYTZD had a significant therapeutic effect on NAFLD-related symptoms. In this study, two chemical components common to SYTZD and rat serum were identified by UPLC-Q/TOF-MS, including diosgenin and stigmasterol. In addition, using network pharmacology analysis to elucidate the complex mechanisms of action of SYTZD, we revealed the multi-component, multi-target, and multi-pathway mechanisms of SYTZD in the treatment of NAFLD. Finally, we constructed HFD-fed NAFLD rat models and FFA-induced high-fat HepG2 cell models for exploring the protective mechanisms of SYTZD against NAFLD. The results of the study showed that SYTZD led to improvements in NAFLD-related indicators, including lipid deposition, insulin resistance, and inflammation. SYTZD may exert multi-anti-NAFLD mechanisms for the treatment of NAFLD. Our findings provide an experimental reference for the clinical use of SYTZD in NAFLD treatment and a new perspective for the study of TCM against NAFLD.

Network pharmacology can explain the interaction mechanisms between components and disease in TCM at the molecular level from a holistic perspective, which provides a theoretical and scientific basis for studying the effectiveness of TCM in treatment of diseases (Guo et al., 2019). Based on the network pharmacology analysis, we identified 8 candidate targets of SYTZD against NAFLD followed by PPI network and MCODE cluster analyses. One cluster and six core genes were obtained, and the core genes included ESR1, mTOR, FASN, HIF-1α, VEGFA, and GSK-3β. The core genes were identified to be involved in the progression of NAFLD, and they might be the key targets for SYTZD in the treatment of NAFLD. To further elucidate the underlying mechanisms, we performed KEGG pathway enrichment analyses of the core targets. KEGG results showed that SYTZD might exert multi-anti-NAFLD effects by activating the thyroid hormone, insulin resistance, HIF-1, mTOR, and AMPK pathways. Finally, the protective effect of SYTZD on NAFLD and the expression levels of the core targets were validated by *in vitro* and *in vivo* experiments. The results revealed that the multi-anti-NAFLD mechanisms of SYTZD might be associated with enhancement of ESR1, p-GSK-3β protein and inhibition of mTOR, FASN, HIF-1α, VEGFA proteins.

Excessive lipid deposition in the liver is a central issue in the development and progression of NAFLD (Lin et al., 2021). As the liver is an important organ in regulating lipid homeostasis, excess fat can accumulate and overload the liver, which leads to a clinicopathological syndrome of inflammation, hepatocellular steatosis, liver fibrosis, and cirrhosis (Zhao et al., 2020). mTOR is a key factor in the regulation of energy metabolism and biosynthesis and plays an important role in lipid metabolism (Yi et al., 2020). mTOR exists as two complexes, mTOR complex 1 and 2 (mTORC1 and mTORC2, respectively). It is reported that mTORC1 regulates lipid metabolic signaling and the balance of systemic homeostasis (Caron et al., 2015). Studies have shown that mTORC1 is involved in the mTOR/SREBP-1c/FASN pathway, which is a key pathway involved in regulation of cellular lipid metabolism and is closely associated with the development of NAFLD (Yi et al., 2020). Sterol-regulatory element binding protein-1c (SREBP-1c) is an important transcriptional regulator in hepatic lipid synthesis, and it is primarily responsible for the expression of key enzymes in fatty acid synthesis, such as FASN (Nguyen et al., 2021). FASN is a key enzyme in the synthesis of fatty acids, which catalyzes acetyl coenzyme A used in fatty acid synthesis (Yang and Mottillo, 2020). Previous studies have shown that FASN overexpression promotes massive accumulation of lipid in hepatocytes and induces oxidative damage of liver tissues, which leads to disorders of lipid and energy metabolism (Ioannou et al., 2019). Additionally, the mTOR signaling pathway is one of the major pathways involved in autophagy regulation (Wang and Zhang, 2019). Several studies have shown that autophagic activity of hepatocytes was promoted through inhibition of mTOR pathway, which alleviates lipid accumulation and inflammation in HFD-fed mice (Park et al., 2021). As a nuclear receptor family, ESR1 is a ligand-activated transcriptional regulator of metabolic processes, including lipid metabolism, bile acid homeostasis, energy expenditure, and hepatitis (Khristi et al., 2019a). An imbalance in these processes can accelerate lipid metabolism disorders. Studies have shown that the ESR1 gene knockout led to weight gain, obesity, and lipid deposition in rats (Khristi et al., 2019b). Our study shows that SYTZD treatment led to a marked reduction in liver lipid deposition, hepatocyte steatosis, and elevated levels of serum TG, TC, and LDL-C in HFD-fed NAFLD rats. SYTZD intervention produced a significant reduction in lipid accumulation and previously elevated levels of intracellular TG and TC in FFA-induced HepG2 cells. Besides, we found an increase in the relative protein levels of p-mTOR and FASN and decrease in the relative protein level of ESR1 in *in vitro and in vivo* models. SYTZD treatment led to a reduction in the levels of p-mTOR and FASN and an increase in the level of ESR1. These results reveal that SYTZD exerts significant lipid-lowing effects. SYTZD might cause a reduction in lipid accumulation by regulating relative protein levels of p-mTOR, FASN, and ESR1 for alleviating NAFLD.

Inflammation is one of the key drivers of the progression of simple fatty liver to steatohepatitis (Rohm et al., 2022). The HIF-1 signaling pathway plays a crucial role in the body inflammation (Korbecki et al., 2021). In NAFLD, liver fat deposits cause swelling of the hepatocytes, cause deformation of liver sinusoids and impairment of microcirculation, and lead to ischemia and hypoxia (Tirosh, 2018). HIF-1α is an important regulator of hypoxia and promotes the transcription and expression of hypoxia-responsive elements in target genes such as VEGFA, thereby regulating hypoxic pre-adaptation of the body (Zhang et al., 2020). It is reported that hypoxia in hepatocytes led to an increased expression of HIF-1α and VEGFA, which further aggravated liver injury as was confirmed in studies of alcoholic fatty liver although minimal research was done in terms of NAFLD (Drygalski et al., 2021). In addition, the release of pro-inflammatory factors, such as IL-1β and TNF-α, can induce liver injury (Shen et al., 2020). When liver damage occurs, significant increases in the levels of serum transaminases, including ALT and AST, were found (McConnell et al., 2021). Our study found that SYTZD treatment led to a significant reduction in the elevated levels of IL-1β, TNF-α, ALT, and AST in HFD-fed NAFLD rats. The results indicate that SYTZD could lead to an effective reversal in HFD-induced inflammation and liver injury. Additionally, we found increased relative protein levels of HIF-1α and VEGFA in *in vitro and in vivo* models. SYTZD treatment led to a reduction in HIF-1α and VEGFA levels. The results reveal that SYTZD might reduce liver inflammation and injury by causing a decrease in the relative protein levels of HIF-1α and VEGFA for alleviating NAFLD.

Insulin resistance is a vital step in the development of NAFLD (Watt et al., 2019). When the body develops insulin resistant, insulin target organs, such as the liver, fat, and muscle, become less sensitive to insulin, which makes it difficult to maintain blood glucose at normal levels (Softic et al., 2020). An elevated blood glucose induces the conversion of glucose into fat, resulting in massive accumulation in the liver, which then triggers NAFLD (Fujii et al., 2020). Therefore, improvement in insulin resistance is an important part of preventing and treating NAFLD. GSK-3β is a key enzyme involved in hepatic glucose metabolism (Zhang et al., 2018). Studies have shown that overexpression of GSK-3β causes hypoactivity of its downstream substrate, glycogen synthase, and synthesis of abnormal glycogen, which leads to insulin resistance (Akhtar and Sah, 2020). Inhibited protein expression of GSK-3β and elevation of p-GSK-3β led to stimulation in glycogen synthesis and an improvement in glucose metabolism disorders, thereby alleviating insulin resistance (Guan et al., 2021). Our study demonstrated that SYTZD treatment significantly reversed the elevated FBG, AUC and HOMA-IR in HFD-fed NAFLD rats. The results reveal that SYTZD caused an improvement in insulin sensitivity and insulin resistance. In addition, we found a decrease in the relative protein level of p-GSK-3β in *in vitro and in vivo* models. SYTZD treatment produced an increase in the level of p-GSK-3β. These results indicate that SYTZD might attenuate insulin resistance by facilitating an increase in the relative protein level of p-GSK-3β for alleviating NAFLD.

Several study limitations should be discussed. The targets predicted through network pharmacology are mostly based on current findings, which limits the discovery of new therapeutic targets. Except for the pharmacologically predicted pathways of lipid synthesis, inflammation, and insulin resistance, SYTZD can lead to attenuation of NAFLD possibly through different mechanisms, such as oxidative stress, mitochondrial dysfunction, intestinal flora imbalance, and genetic factors. The exact pharmacological mechanisms of SYTZD in the treatment of NAFLD should be explored in the future. Additionally, our study explored the dose-dependent effects of SYTZD on NAFLD using only two concentrations. Further research should include establishment of concentration gradient of SYTZD for exploring the dose-effect relationship.

## Conclusion

In conclusion, we explored the potential targets and molecular mechanisms for SYTZD against NAFLD based on network pharmacological analysis. ESR1, mTOR, FASN, HIF-1α, VEGFA, and GSK-3β might be potential therapeutic targets for SYTZD in the treatment of NAFLD. Furthermore, experiments *in vitro* and *in vivo* indicate that SYTZD might be involved in multi-anti-NAFLD mechanisms, including improvements in lipid deposition, inflammation, and insulin resistance. SYTZD treatment affected relative mRNA and protein levels of mTOR, FASN, ESR1, HIF-1α, VEGFA, and p-GSK-3β. However, the underlying mechanisms of above genes in SYTZD treatment of NAFLD should be tested in the NAFLD model in the future. Our findings provide scientific evidence to support the clinical application of SYTZD in the treatment of NAFLD and provide a reliable reference for exploring the pharmacological mechanisms of SYTZD action.

## Data Availability

The original contributions presented in the study are included in the article/Supplementary Material, further inquiries can be directed to the corresponding author.

## Glossary

NAFLD: non-alcoholic fatty liver disease
SYTZD: Shuangyu Tiaozhi Decoction
UPLC-Q/TOF-MS: ultra-performance liquid chromatography to quadrupole/time-of-flight mass spectrometry
TCMSP: traditional Chinese medical systems pharmacology
ETCM: encyclopedia of traditional Chinese medicine
PPI: protein–protein interaction
KEGG: Kyoto Encyclopedia of Genes and Genomes
DAVID: database for annotation, visualization, and integrated discovery
FDA: Food and Drug Administration
TCM: traditional Chinese medicine
OB: oral availability
DL: drug-likeness
HFD: High-fat diets
FBG: fasting blood glucose
AUC: area under the curve
FINS: fasting insulin
ELISA: enzyme-linked immunosorbent assay
HOMA-IR: homeostasis model assessment insulin resistance
TC: total cholesterol
TG: triglycerides
LDL-C: low-density lipoprotein cholesterol
ALT: alanine aminotransferase
AST: aspartate aminotransferase
IL-1β: interleukin 1 beta
TNF-α: tumor necrosis alpha
H&E: hematoxylin and eosin
FFAs: free fatty acids
CCK-8: cell counting kit-8
RT-qPCR: real-time quantitative polymerase chain reaction
GAPDH: glyceraldehyde-3-phosphate dehydrogenase
WB: western blotting
SDS-PAGE: sodium dodecyl sulfate polyacrylamide gel electrophoresis
ESR1: estrogen receptor 1
mTOR: mammalian target of rapamycin
p-mTOR: phospho-mammalian target of rapamycin
FASN: fatty acid synthase
HIF-1α: hypoxia-inducible factor-1α
VEGFA: vascular endothelial growth factor-A
GSK-3β: glycogen synthase kinase-3 beta
p-GSK-3β: phospho-glycogen synthase kinase-3 beta
HRP: horseradish peroxidase
ANOVA: one-way analysis of variance
mTORC1: mTOR complex 1
mTORC2: mTOR complex 2
SREBP-1c: sterol-regulatory element binding protein-1c
