# Supplementary figures and images for: Shuangyu Tiaozhi decoction alleviates non-alcoholic fatty liver disease by improving lipid deposition, insulin resistance, and inflammation in vitro and in vivo
Source: Front Pharmacol. 2022 Nov 23;13:1016745. doi: 10.3389/fphar.2022.1016745 (PMC9727266; doi:10.3389/fphar.2022.1016745)

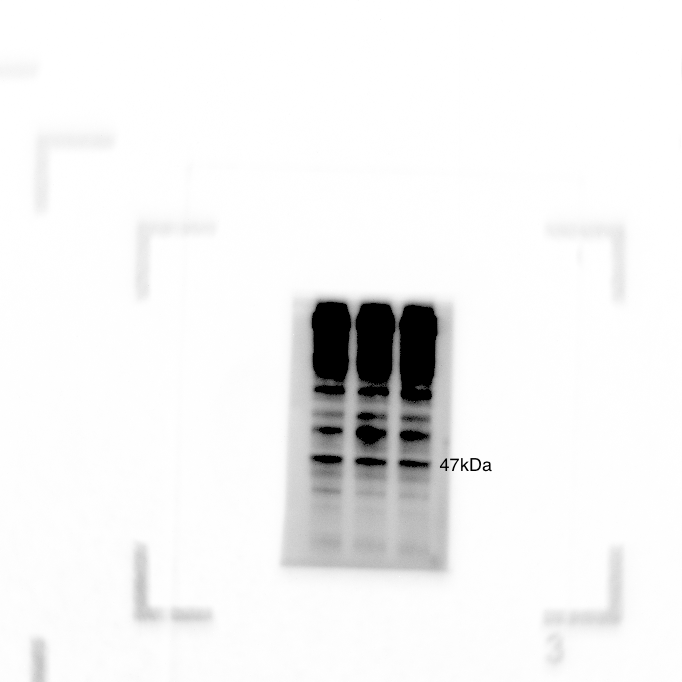

Supplement: Supplementary file 1 [file DataSheet1.ZIP › Original Image for western blots/Original Image for Fig 12L_GSK3╬▓.tif]

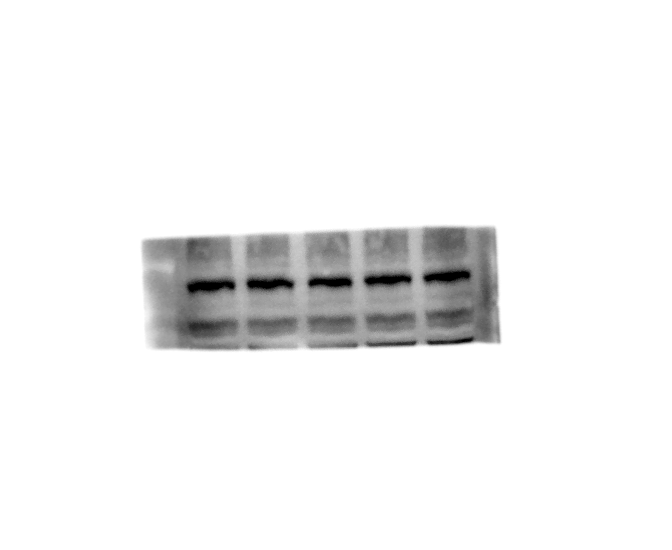

Supplement: Supplementary file 1 [file DataSheet1.ZIP › Original Image for western blots/Original Image for Fig 9F_GSK-3╬▓.tif]

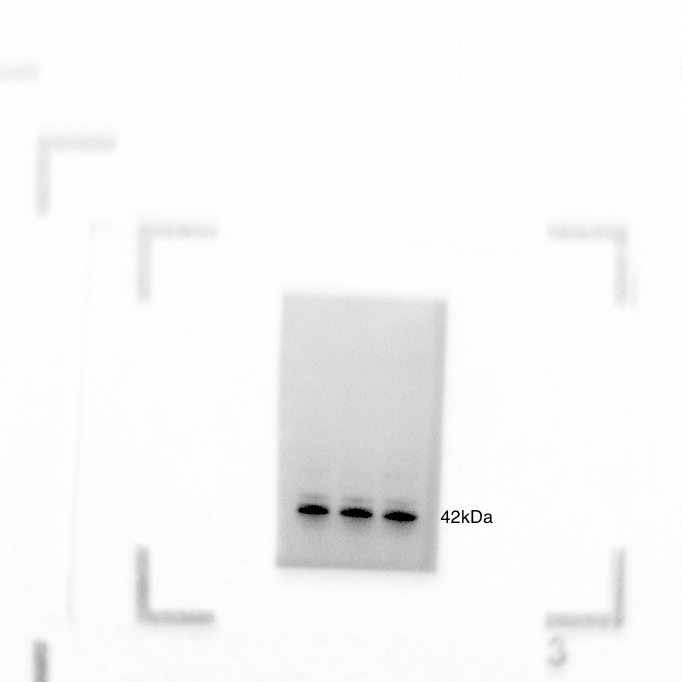

Supplement: Supplementary file 1 [file DataSheet1.ZIP › Original Image for western blots/Original Image for Fig 12H_FASN_╬▓_actin.tif]

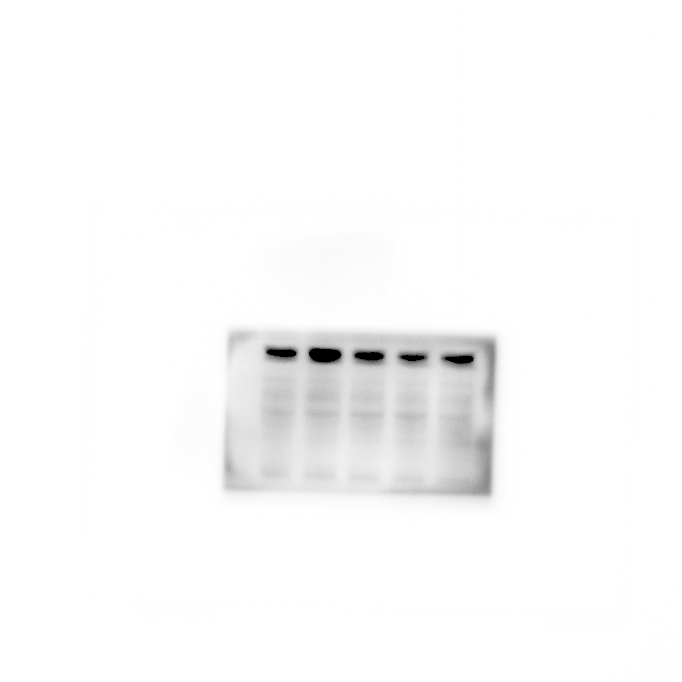

Supplement: Supplementary file 1 [file DataSheet1.ZIP › Original Image for western blots/Original Image for Fig 9D_HIF1╬▒.tif]

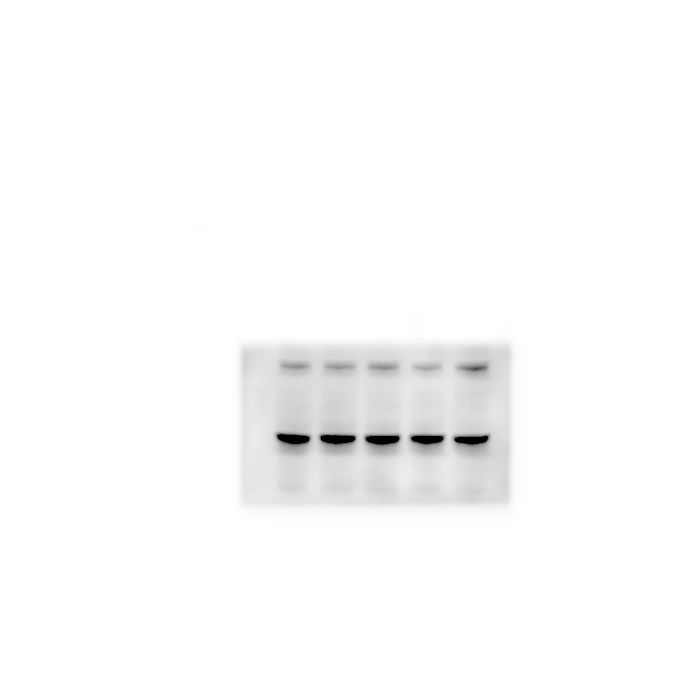

Supplement: Supplementary file 1 [file DataSheet1.ZIP › Original Image for western blots/Original Image for Fig 9D_HIF1╬▒_╬▓_actin.tif]

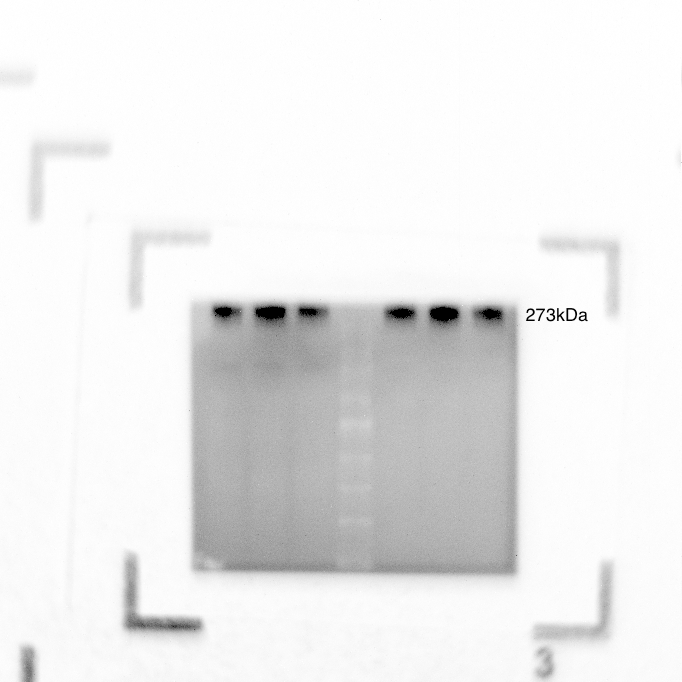

Supplement: Supplementary file 1 [file DataSheet1.ZIP › Original Image for western blots/Original Image for Fig 12H_FASN.tif]

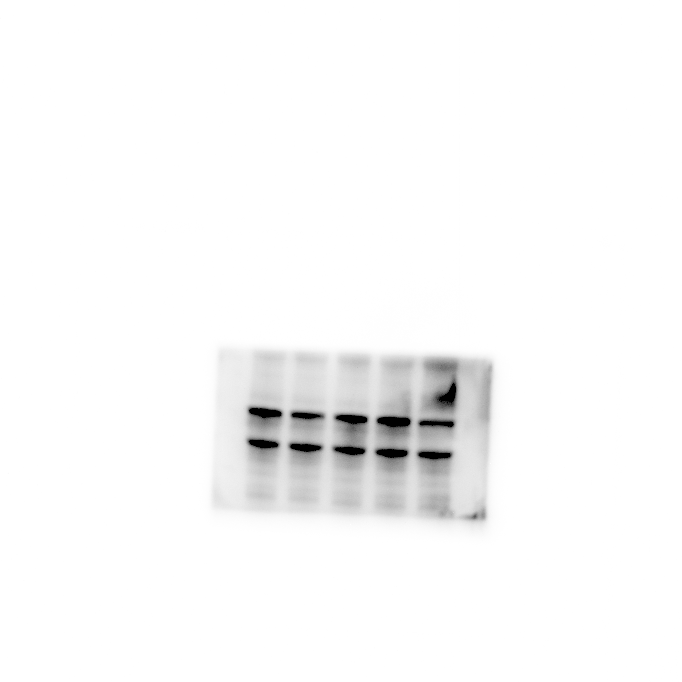

Supplement: Supplementary file 1 [file DataSheet1.ZIP › Original Image for western blots/Original Image for Fig 9C_ESR1_╬▓_actin.tif]

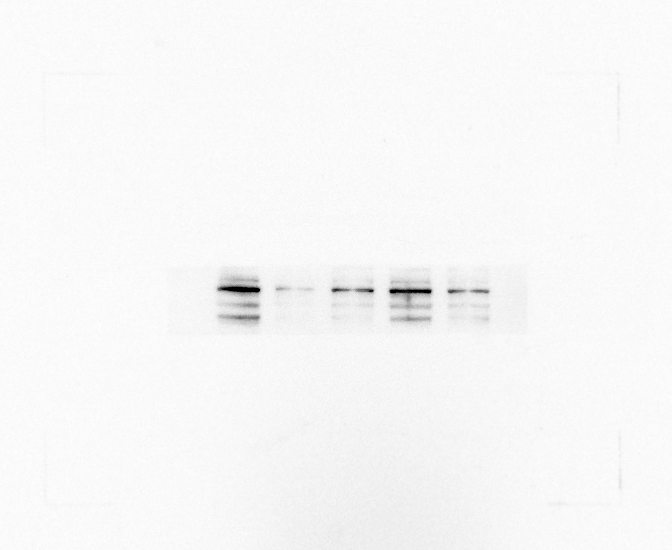

Supplement: Supplementary file 1 [file DataSheet1.ZIP › Original Image for western blots/Original Image for Fig 9F_pGSK-3╬▓.tif]

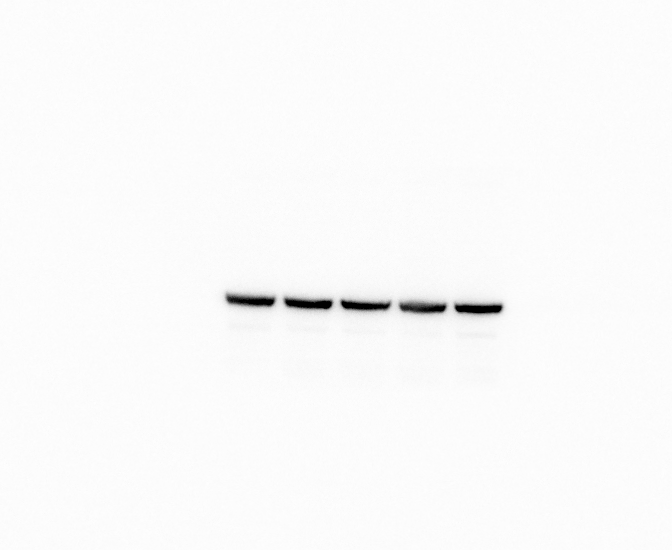

Supplement: Supplementary file 1 [file DataSheet1.ZIP › Original Image for western blots/Original Image for Fig 9E_VEGFA_╬▓_actin.tif]

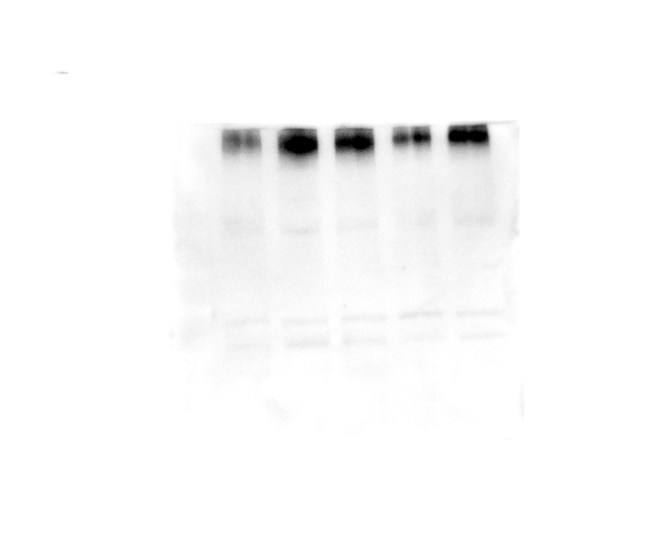

Supplement: Supplementary file 1 [file DataSheet1.ZIP › Original Image for western blots/Original Image for Fig 9B_FASN.tif]

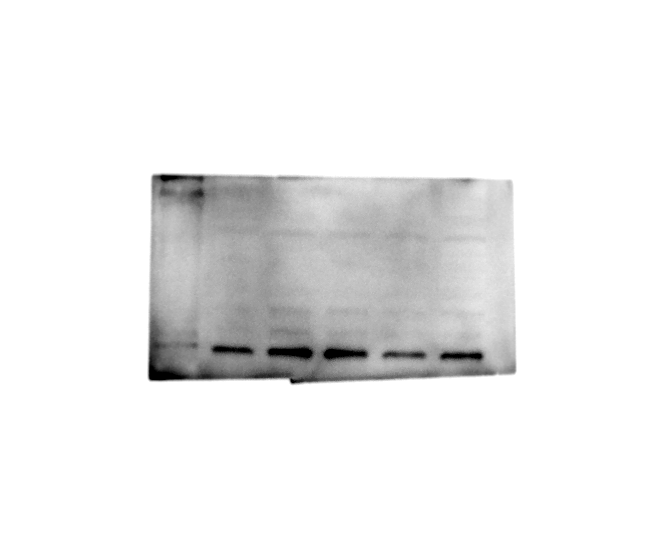

Supplement: Supplementary file 1 [file DataSheet1.ZIP › Original Image for western blots/Original Image for Fig 9E_VEGFA.tif]

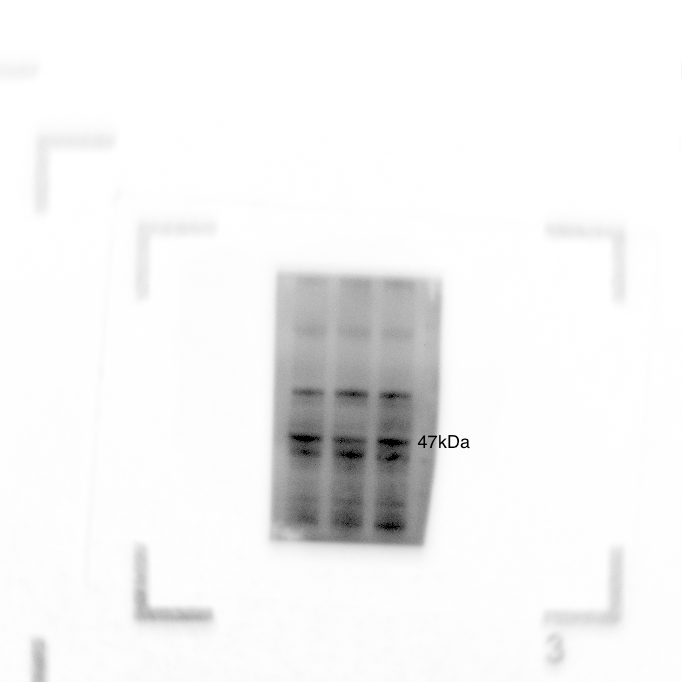

Supplement: Supplementary file 1 [file DataSheet1.ZIP › Original Image for western blots/Original Image for Fig 12L_p_GSK3╬▓.tif]

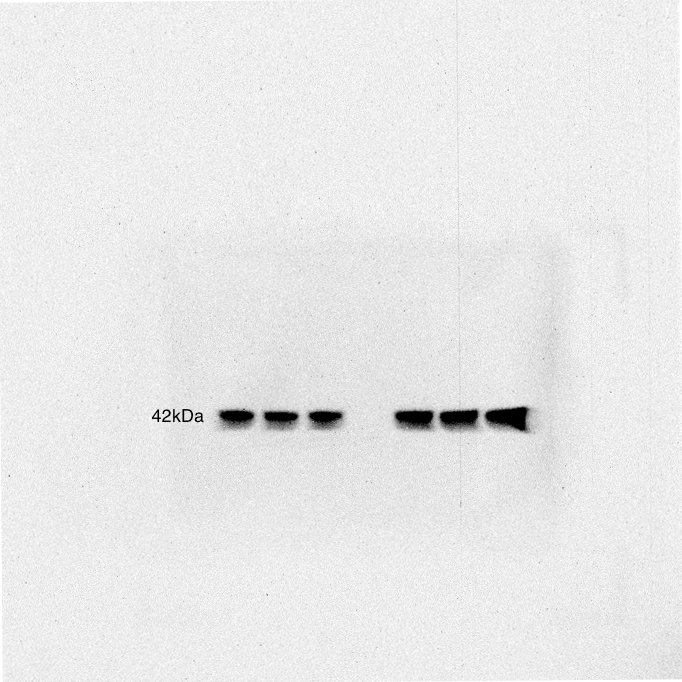

Supplement: Supplementary file 1 [file DataSheet1.ZIP › Original Image for western blots/Original Image for Fig 12K_VEGFA_╬▓_actin.tif]

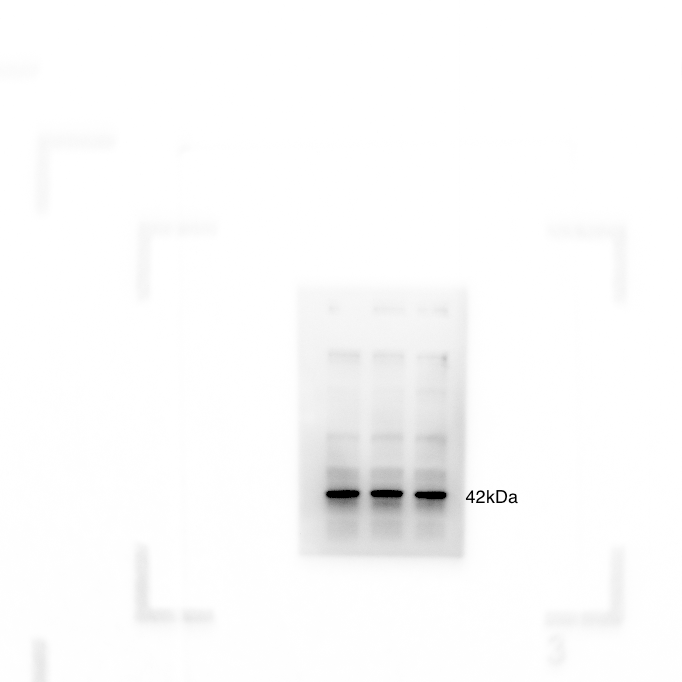

Supplement: Supplementary file 1 [file DataSheet1.ZIP › Original Image for western blots/Original Image for Fig 12G_p_mTOR_╬▓_actin.tif]

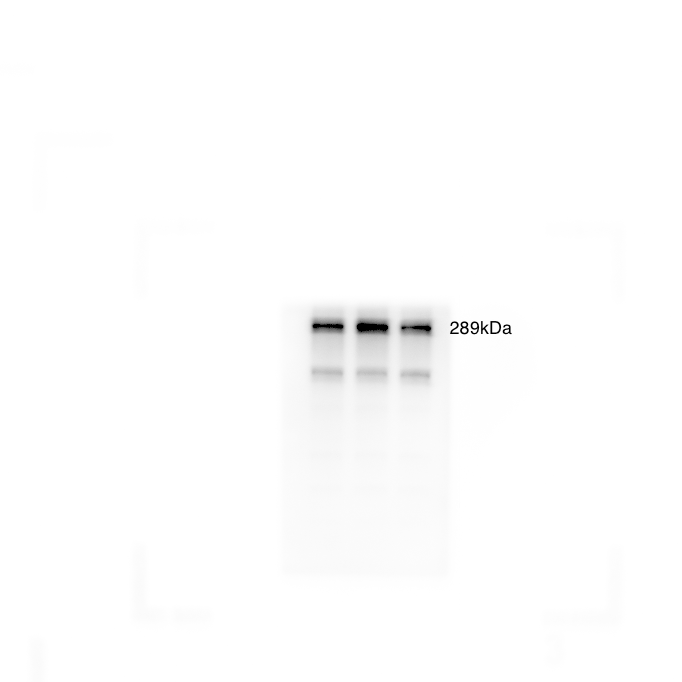

Supplement: Supplementary file 1 [file DataSheet1.ZIP › Original Image for western blots/Original Image for Fig 12G_p_mTOR.tif]

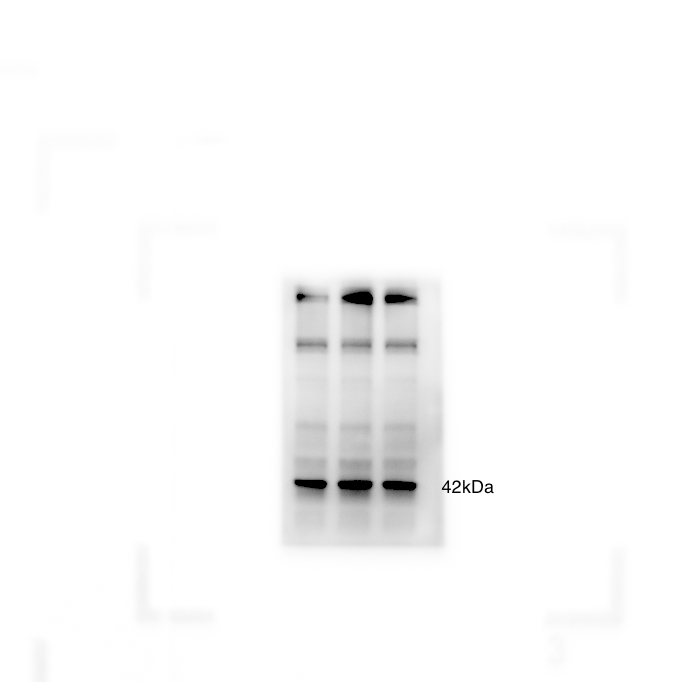

Supplement: Supplementary file 1 [file DataSheet1.ZIP › Original Image for western blots/Original Image for Fig 12L_GSK3╬▓_╬▓_actin.tif]

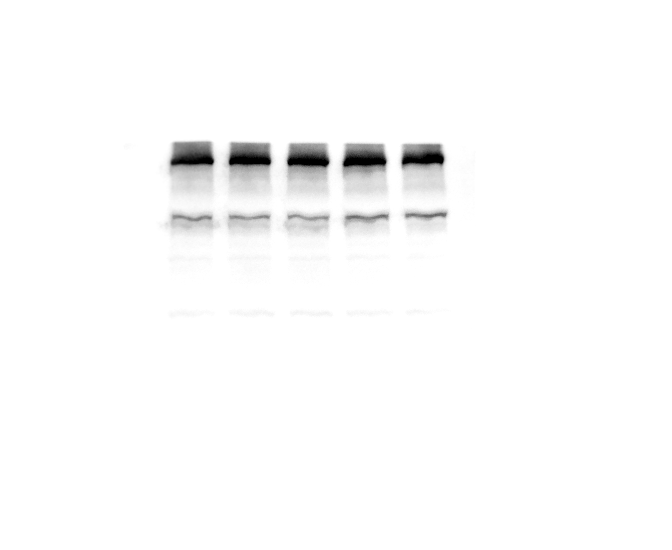

Supplement: Supplementary file 1 [file DataSheet1.ZIP › Original Image for western blots/Original Image for Fig 9A_mTOR.tif]

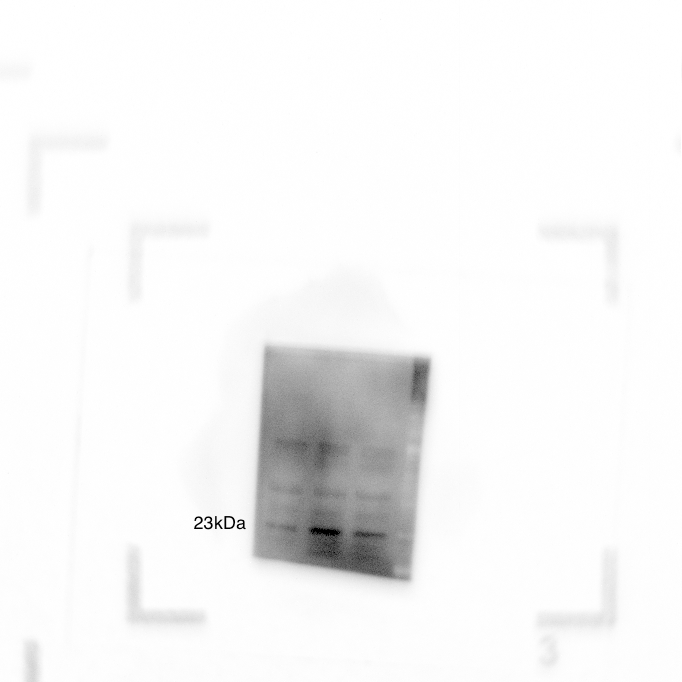

Supplement: Supplementary file 1 [file DataSheet1.ZIP › Original Image for western blots/Original Image for Fig 12K_VEGFA.tif]

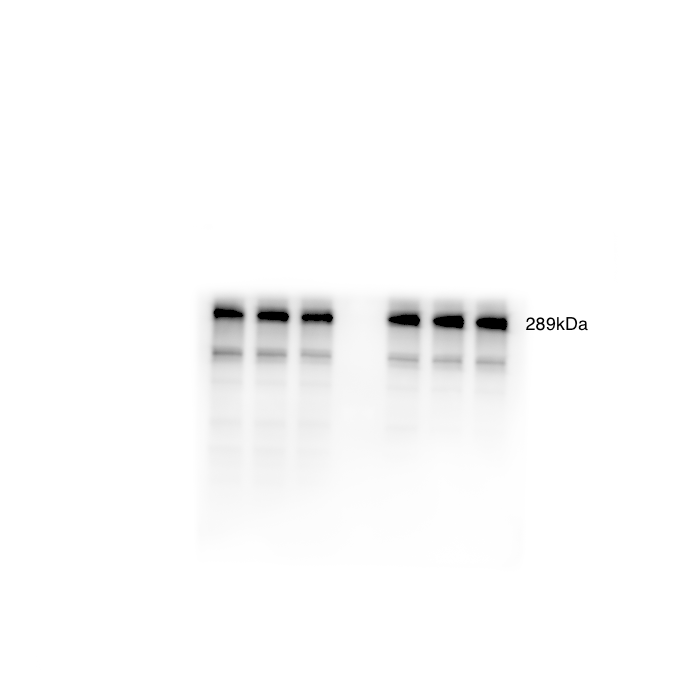

Supplement: Supplementary file 1 [file DataSheet1.ZIP › Original Image for western blots/Original Image for Fig 12G_mTOR.tif]

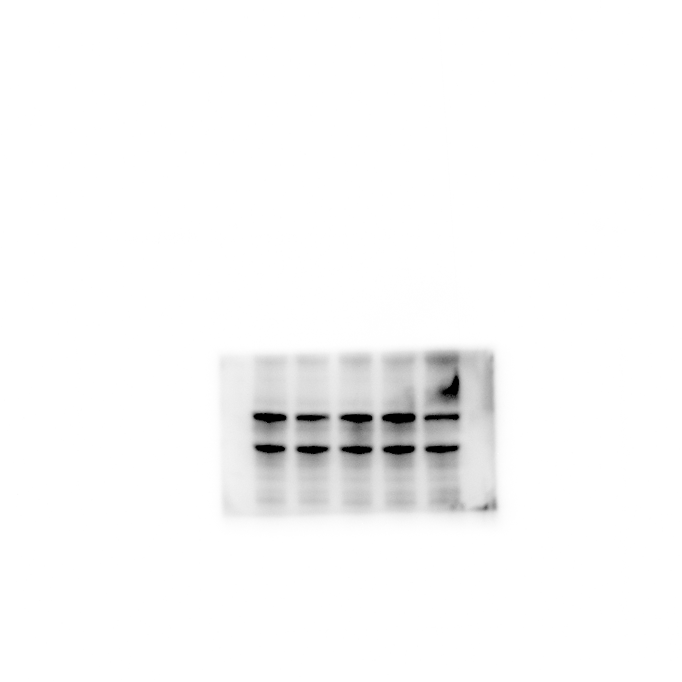

Supplement: Supplementary file 1 [file DataSheet1.ZIP › Original Image for western blots/Original Image for Fig 9C_ESR1.tif]

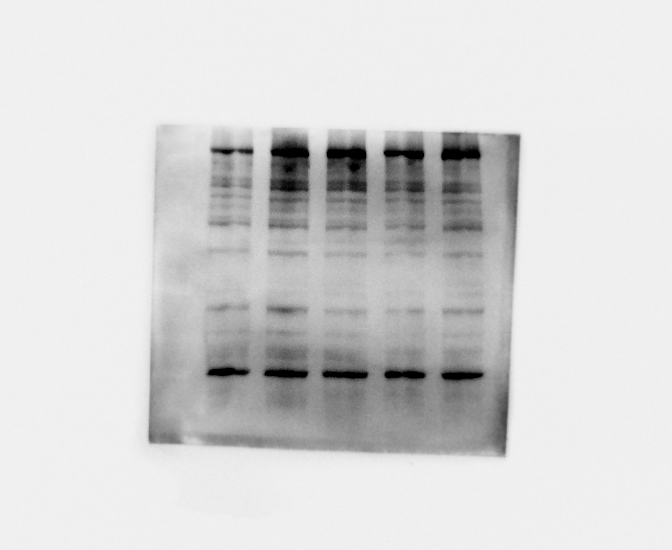

Supplement: Supplementary file 1 [file DataSheet1.ZIP › Original Image for western blots/Original Image for Fig 9A_p_mTOR_╬▓_actin.tif]

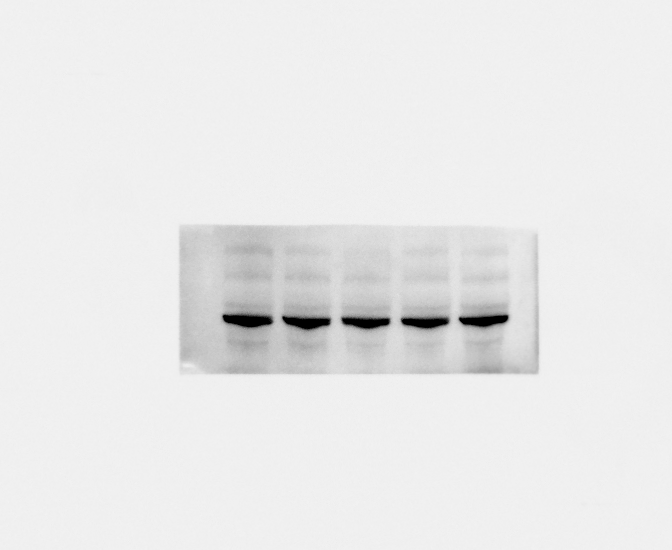

Supplement: Supplementary file 1 [file DataSheet1.ZIP › Original Image for western blots/Original Image for Fig 9F_GSK-3╬▓_╬▓_actin.tif]

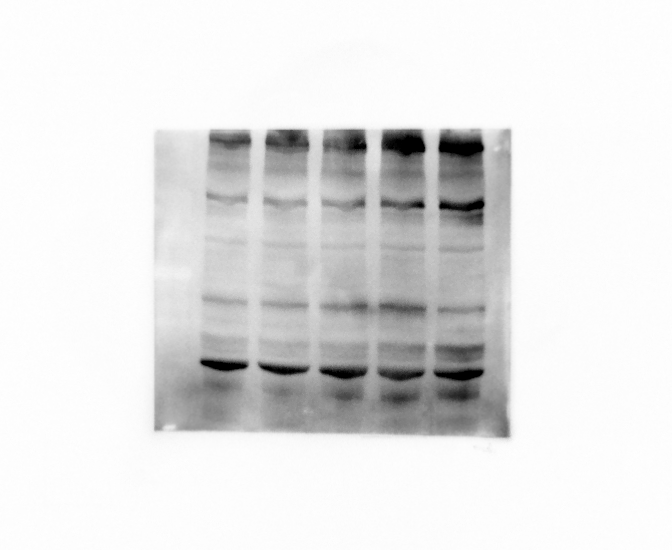

Supplement: Supplementary file 1 [file DataSheet1.ZIP › Original Image for western blots/Original Image for Fig 9B_FASN_╬▓_actin.tif]

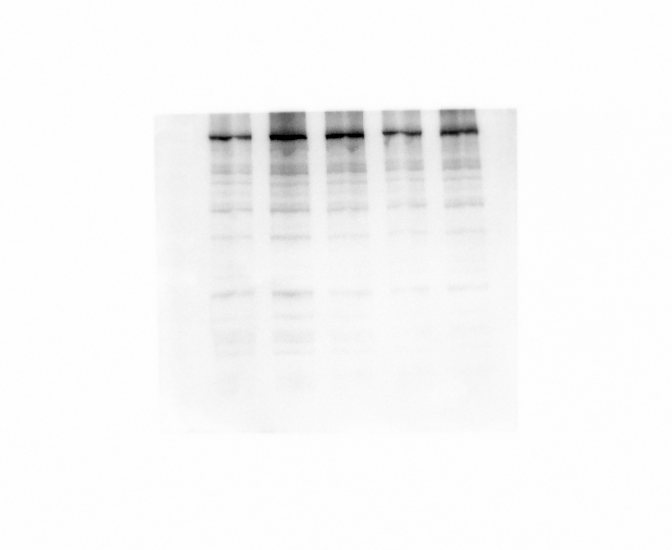

Supplement: Supplementary file 1 [file DataSheet1.ZIP › Original Image for western blots/Original Image for Fig 9A_p_mTOR.tif]

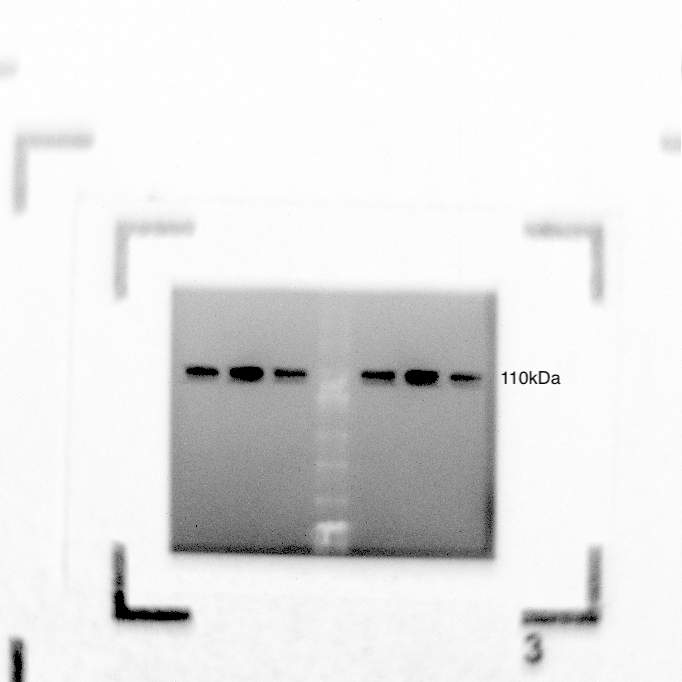

Supplement: Supplementary file 1 [file DataSheet1.ZIP › Original Image for western blots/Original Image for Fig 12J_HIF1╬▒.tif]

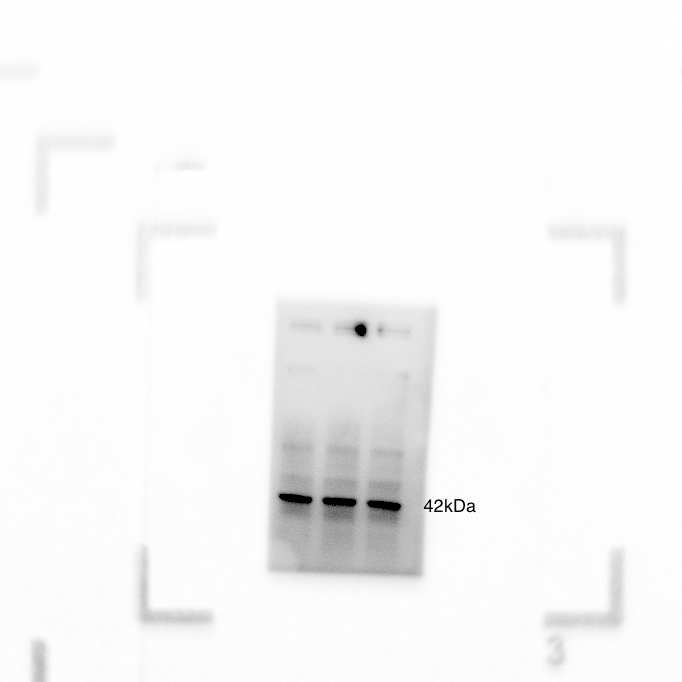

Supplement: Supplementary file 1 [file DataSheet1.ZIP › Original Image for western blots/Original Image for Fig 12I_ESR1_╬▓_actin.tif]

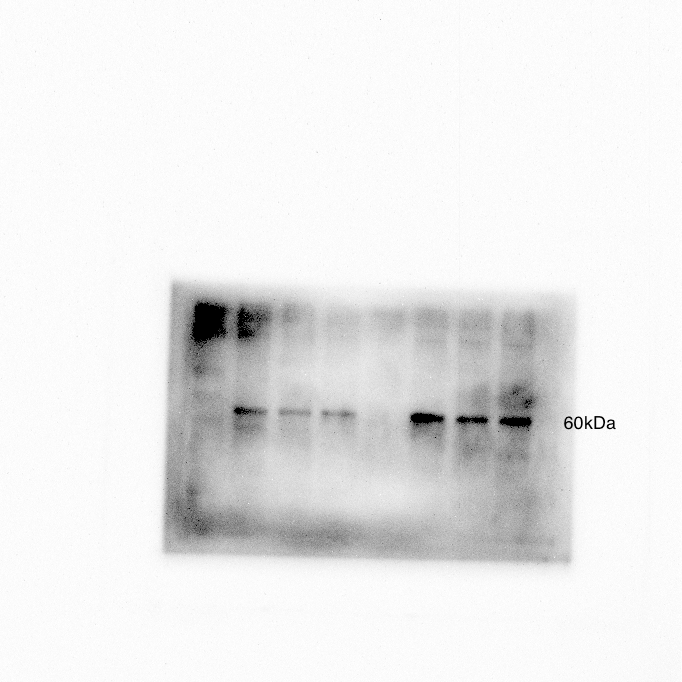

Supplement: Supplementary file 1 [file DataSheet1.ZIP › Original Image for western blots/Original Image for Fig 12I_ESR1.tif]

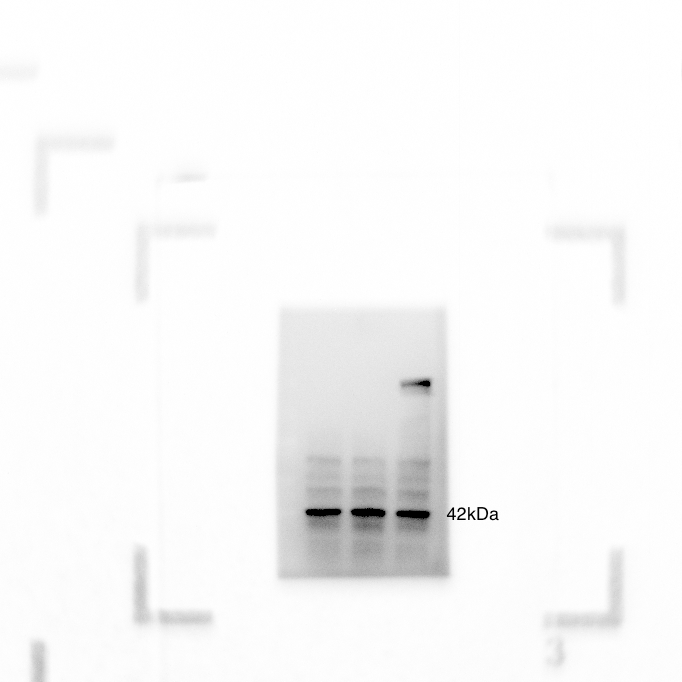

Supplement: Supplementary file 1 [file DataSheet1.ZIP › Original Image for western blots/Original Image for Fig 12J_HIF1╬▒_╬▓_actin.tif]
