# Supplementary material for: Shuangyu Tiaozhi decoction alleviates non-alcoholic fatty liver disease by improving lipid deposition, insulin resistance, and inflammation in vitro and in vivo
Source: Front Pharmacol. 2022 Nov 23;13:1016745. doi: 10.3389/fphar.2022.1016745 (PMC9727266; doi:10.3389/fphar.2022.1016745)
Supplement: Supplementary file 2 [file DataSheet2.DOCX]

Supplementary Material

# Supplementary Tables

Supplementary Table S1. Mass spectrometry data and elemental composition of compounds in SYTZD by UPLC-Q/TOF-MS analysis

| **Name** | **Formula** | **Mass (Da)** | **RT (min)** | **Area** |
| --- | --- | --- | --- | --- |
| Positive ion mode | |  |  |  |
| Guanosine | C10H13N5O5 | 283.0917 | 1.77 | 10156 |
| Leu | C6H13NO2 | 131.0946 | 0.87 | 168757 |
| L-Tryptophan | C11H12N2O2 | 204.0899 | 2.49 | 155979 |
| Nicotinic acid | C6H5NO2 | 123.0320 | 0.86 | 14048 |
| Scopolamine | C17H21NO4 | 303.1471 | 0.73 | 11600 |
| Cordycepin | C10H13N5O3 | 251.1018 | 1.97 | 6005 |
| Adenosine | C10H13N5O4 | 267.0967 | 1.95 | 145669 |
| L-Tyrosine | C9H11NO3 | 181.0739 | 0.82 | 102922 |
| Tyr | C9H11NO3 | 181.0739 | 0.82 | 102916 |
| Coproporphyrin I | C36H38N4O8 | 654.2690 | 3.24 | 3446 |
| Niacinamide | C6H6N2O | 122.0480 | 1.64 | 17203 |
| Tuberostemonine | C22H33NO4 | 375.2410 | 8.14 | 1577 |
| 5''-Methylthioadenosine | C11H15N5O3S | 297.0896 | 2.83 | 15157 |
| Thr | C4H9NO3 | 119.0582 | 1.96 | 6530 |
| Stigmasterol | C29H48O | 412.6950 | 5.69 | 8095 |
| Phellodendrine | C20H23NO4 | 341.1627 | 3.27 | 15153 |
| Deoxyadenosine | C10H13N5O3 | 251.1018 | 1.97 | 6005 |
| Pantothenic acid | C9H17NO5 | 219.1107 | 2.09 | 31164 |
| Pyroglutamic acid | C5H7NO3 | 129.0426 | 0.76 | 25562 |
| Pyrrolidonecarboxylic acid | C5H7NO3 | 129.0426 | 0.76 | 25562 |
| Apigenin 7-O-beta-D-glucuronide | C21H18O11 | 446.0849 | 4.90 | 1451 |
| Syringaldehyde | C9H10O4 | 182.0579 | 4.18 | 3447 |
| 2-Piperidinone | C5H9NO | 99.0684 | 2.12 | 2453 |
| L-Valine | C5H11NO2 | 117.0790 | 0.73 | 59443 |
| Adenine | C5H5N5 | 135.0545 | 1.08 | 68795 |
| Phenylpropanolamine | C9H13NO | 151.0997 | 0.75 | 1639 |
| Riboflavin | C17H20N4O6 | 376.1383 | 3.41 | 3366 |
| Diosgenin | C27H42O3 | 414.3134 | 8.72 | 117571 |
| L-Carnitine | C7H15NO3 | 161.1052 | 0.69 | 25197 |
| 4-Guanidinobutanoic acid | C5H11N3O2 | 145.0851 | 0.72 | 57240 |
| L-Norleucine | C6H13NO2 | 131.0946 | 0.87 | 168758 |
| Pyridoxine | C8H11NO3 | 169.0739 | 0.96 | 5480 |
| Indole | C8H7N | 117.0578 | 2.49 | 7773 |
| Pipecolic acid | C6H11NO2 | 129.0790 | 0.73 | 74987 |
| 4'-Hydroxyacetophenone | C8H8O2 | 136.0524 | 0.96 | 11619 |
| Anisaldehyde | C8H8O2 | 136.0524 | 0.96 | 11619 |
| Epimedin A | C39H50O20 | 884.2950 | 5.28 | 117 |
| Guanine | C5H5N5O | 151.0494 | 1.77 | 15099 |
| Cinnamaldehyde | C9H8O | 132.0575 | 5.57 | 10934 |
| Gamma-Aminobutyric acid | C4H9NO2 | 103.0633 | 0.69 | 6278 |
| Polyphyllin A | C39H62O12 | 722.4241 | 8.72 | 38060 |
| 3-Hexenedioic acid | C6H8O4 | 144.0423 | 1.90 | 22149 |
| Methyl linoleate | C19H34O2 | 294.2559 | 12.52 | 35121 |
| L-Alloisoleucine | C6H13NO2 | 131.0946 | 0.87 | 168758 |
| Indoleacrylic acid | C11H9NO2 | 187.0633 | 2.49 | 300785 |
| L-Isoleucine | C6H13NO2 | 131.0946 | 0.87 | 168758 |
| Secoisolariciresinol diglucoside | C32H46O16.NH3 | 703.3051 | 3.16 | 5499 |
| Kadsurenone | C21H24O5 | 356.3917 | 7.24 | 1064 |
| Neotuberostemonine | C22H33NO4 | 375.2410 | 8.14 | 1577 |
| Trigonelline | C7H7NO2 | 137.0477 | 0.70 | 179127 |
| L-Arginine | C6H14N4O2 | 174.1117 | 0.66 | 290936 |
| Vitamin B2 | C17H20N4O6 | 376.1383 | 3.41 | 3366 |
| Hydroxyisocaproic acid | C6H12O3 | 132.0786 | 2.79 | 4922 |
| Piperine | C17H19NO3 | 285.1365 | 7.98 | 1495 |
| Menadione | C11H8O2 | 172.0524 | 5.53 | 7793 |
| Camphor | C10H16O | 152.1201 | 12.06 | 1282 |
| Phenylglyoxylic acid | C8H6O3 | 150.0317 | 4.90 | 2136 |
| Schaftoside | C26H28O14 | 564.1479 | 3.59 | 3341 |
| Imidazoleacetic acid | C5H6N2O2 | 126.0429 | 2.21 | 4862 |
| Vitamin B6 | C8H11NO3 | 169.0739 | 0.96 | 5480 |
| 3-Aminoisobutanoic acid | C4H9NO2 | 103.0633 | 0.69 | 6278 |
| Citramalic acid | C5H8O5 | 148.0372 | 5.54 | 3261 |
| Emodin | C15H10O5 | 270.0528 | 4.48 | 4933 |
| Tryptamine | C10H12N2 | 160.1001 | 7.55 | 2935 |
| Kynurenine | C10H12N2O3 | 208.0848 | 4.79 | 1623 |
| Fragransin A2 | C20H24O5 | 344.1624 | 5.74 | 10603 |
| His | C6H9N3O2 | 155.0695 | 0.68 | 13551 |
| o-Tyrosine | C9H11NO3 | 181.0739 | 0.82 | 102922 |
| 6-Methylcoumarin | C10H8O2 | 160.0524 | 3.98 | 14445 |
| 3-Methylindole | C9H9N | 131.0735 | 2.49 | 5760 |
| Histamine | C5H9N3 | 111.0797 | 0.75 | 32275 |
| Stearic acid | C18H36O2 | 284.2715 | 12.16 | 5881 |
| Arg | C6H14N4O2 | 174.1117 | 0.66 | 290940 |
| 4-Hydroxyproline | C5H9NO3 | 131.0582 | 0.75 | 617 |
| Picolinic acid | C6H5NO2 | 123.0320 | 0.86 | 14048 |
| Homo-L-arginine | C7H16N4O2 | 188.1273 | 0.69 | 23317 |
| trans-Cinnamic acid | C9H8O2 | 148.0524 | 1.96 | 4869 |
| Linderane | C15H16O4 | 260.1049 | 7.80 | 3993 |
| 2-Furoic acid | C5H4O3 | 112.0160 | 1.33 | 2085 |
| 3-N-butyl-4,5-dihydrophthalide | C12H16O2 | 192.1150 | 11.98 | 452 |
| Pipecolinic acid | C6H11NO2 | 129.0790 | 0.73 | 74987 |
| 3-Pyridylacetic acid | C7H7NO2 | 137.0477 | 0.70 | 179127 |
| p-Aminobenzoic acid | C7H7NO2 | 137.0477 | 0.70 | 179127 |
| 3-Hydroxyanthranilic acid | C7H7NO3 | 153.0426 | 1.33 | 3221 |
| Negative ion mode | |  |  |  |
| Azelaic acid | C9H16O4 | 188.1049 | 4.68 | 92761 |
| N-Alpha-acetyllysine | C8H16N2O3 | 188.1161 | 4.68 | 19492 |
| Creatine | C4H9N3O2 | 131.0695 | 0.87 | 3276 |
| Dioscin | C45H72O16 | 914.4875 | 8.71 | 294783 |
| Guanosine | C10H13N5O5 | 283.0917 | 1.78 | 12700 |
| Arg | C6H14N4O2 | 174.1117 | 0.64 | 14522 |
| Shikimic acid | C7H10O5 | 174.0528 | 0.72 | 12928 |
| Ile | C6H13NO2 | 131.0946 | 0.88 | 36650 |
| Leu | C6H13NO2 | 131.0946 | 0.88 | 36650 |
| N-Acetyl-L-alanine | C5H9NO3 | 131.0582 | 0.83 | 10315 |
| Pipecolic acid | C6H11NO2 | 129.0790 | 1.03 | 437 |
| Pipecolinic acid | C6H11NO2 | 129.0790 | 1.03 | 437 |
| Pyroglutamic acid | C5H7NO3 | 129.0426 | 0.76 | 289484 |
| Pyrrolidonecarboxylic acid | C5H7NO3 | 129.0426 | 0.76 | 289484 |
| Urea | CH4N2O | 60.0324 | 0.74 | 8013 |
| Succinic acid | C4H6O4 | 118.0266 | 0.93 | 229079 |
| Dimethylmalonic acid | C5H8O4 | 132.0423 | 1.88 | 5842 |
| Isochlorogenic acid B | C25H24O12 | 516.1268 | 4.25 | 4354 |
| Maleic acid | C4H4O4 | 116.0110 | 0.86 | 312465 |
| Tyr | C9H11NO3 | 181.0739 | 1.20 | 52435 |
| Undecanedioic acid | C11H20O4 | 216.1362 | 6.02 | 13195 |
| Malic acid | C4H6O5 | 134.0215 | 0.70 | 635822 |
| 2,3-Dihydroxybenzoic acid | C7H6O4 | 154.0266 | 2.35 | 131568 |
| Protocatechuic acid | C7H6O4 | 154.0266 | 2.35 | 131568 |
| Adenosine 2'',3''-cyclic phosphate | C10H12N5O6P | 329.0525 | 1.92 | 5052 |
| Fumaric acid | C4H4O4 | 116.0110 | 0.86 | 312465 |
| Pantothenic acid | C9H17NO5 | 219.1107 | 2.10 | 15989 |
| Methylsuccinic acid | C5H8O4 | 132.0423 | 1.88 | 5842 |
| Gentisic acid | C7H6O4 | 154.0266 | 2.35 | 131568 |
| 4'-Hydroxyacetophenone | C8H8O2 | 136.0524 | 4.01 | 20215 |
| Eleutheroside E | C34H46O18 | 788.2739 | 3.65 | 9944 |
| Liriopesides B | C39H62O12 | 768.4296 | 9.15 | 76261 |
| L-Tryptophan | C11H12N2O2 | 204.0899 | 2.49 | 80250 |
| L-Malic acid | C4H6O5 | 134.0215 | 0.70 | 635822 |
| 2-Isopropylmalic acid | C7H12O5 | 176.0685 | 2.66 | 131626 |
| Allantoic acid | C4H8N4O4 | 176.0546 | 2.67 | 47264 |
| Erythritol | C4H10O4 | 122.0579 | 3.73 | 495 |
| p-Hydroxybenzaldehyde | C7H6O2 | 122.0368 | 3.72 | 212265 |
| Gingerglycolipid B | C33H58O14 | 724.3881 | 9.01 | 2061 |
| Gallic acid | C7H6O5 | 170.0215 | 1.36 | 85098 |
| Apigenin 7-O-beta-D-glucuronide | C21H18O11 | 446.0849 | 4.90 | 1976 |
| Methylmalonic acid | C4H6O4 | 118.0266 | 0.93 | 229079 |
| 2-Pyrocatechuic acid | C7H6O4 | 154.0266 | 2.35 | 131568 |
| Momordin Ic | C41H64O13 | 764.4347 | 4.88 | 1477 |
| D-Arginine | C6H14N4O2 | 174.1117 | 0.64 | 14522 |
| Hydroxytyrosol | C8H10O3 | 154.0630 | 2.47 | 1831 |
| hancinone C | C23H28O6 | 400.5268 | 4.25 | 4354 |
| Citric acid | C6H8O7 | 192.0270 | 0.74 | 107390 |
| Scopoletin | C10H8O4 | 192.0423 | 0.72 | 39779 |
| L-Glutamic acid | C5H9NO4 | 147.0532 | 0.64 | 5705 |
| Neochlorogenic acid | C16H18O9 | 354.0951 | 2.42 | 26359 |
| Baicalin | C21H18O11 | 446.0849 | 4.90 | 1976 |
| Cyclic AMP | C10H12N5O6P | 329.0525 | 1.92 | 5052 |
| Stearic acid | C18H36O2 | 284.2715 | 12.26 | 26783 |
| Adenine | C5H5N5 | 135.0545 | 1.13 | 6138 |
| Salicylic acid | C7H6O3 | 138.0317 | 3.05 | 40512 |
| 4-Hydroxybenzoic acid | C7H6O3 | 138.0317 | 3.05 | 40512 |
| Gracillin | C45H72O17 | 930.4824 | 6.19 | 106812 |
| Glutaric acid | C5H8O4 | 132.0423 | 1.88 | 5842 |
| Traumatic acid | C12H20O4 | 228.1362 | 6.31 | 30307 |
| Troxerutin | C33H42O19 | 742.2321 | 3.97 | 2258 |
| Quinic acid | C7H12O6 | 192.0634 | 0.64 | 131810 |
| Indolelactic acid | C11H11NO3 | 205.0739 | 4.26 | 1269 |
| Nervonic acid | C24H46O2 | 366.3498 | 12.07 | 1703 |
| N-Acetyl-L-phenylalanine | C11H13NO3 | 207.0895 | 3.65 | 35505 |
| Secoisolariciresinol diglucoside | C32H46O16 | 732.2841 | 3.16 | 7299 |
| 4,5-DCQA Isochlorogenic acid C | C25H24O12 | 516.1268 | 4.25 | 4354 |
| 3-Hydroxybenzoic acid | C7H6O3 | 138.0317 | 3.05 | 40512 |
| 3,5-Dicaffeoyl quinic acid | C25H24O12 | 516.1268 | 4.25 | 4354 |
| His | C6H9N3O2 | 155.0695 | 0.63 | 7142 |
| Succinylacetone | C7H10O4 | 158.0579 | 3.19 | 9290 |
| D-Tagatose | C6H12O6 | 180.0634 | 0.68 | 62256 |
| N-Acetylornithine | C7H14N2O3 | 174.1004 | 0.64 | 11632 |
| 2-Phenylbutyric acid | C10H12O2 | 164.0837 | 5.61 | 1729 |
| Licoagroside D | C22H24O10 | 448.1369 | 4.81 | 7142 |
| Urocanic acid | C6H6N2O2 | 138.0429 | 3.05 | 7935 |
| trans-Aconitic acid | C6H6O6 | 174.0164 | 0.87 | 22924 |
| 1-Methylguanine | C6H7N5O | 165.0651 | 1.88 | 29782 |
| Phenprobamate | C9H11NO2 | 165.0790 | 1.88 | 77564 |
| Uridine | C9H12N2O6 | 244.0695 | 1.35 | 16321 |
| Isoschaftoside | C26H28O14 | 564.1479 | 3.60 | 5527 |
| Suberic acid | C8H14O4 | 174.0892 | 3.97 | 5020 |
| 6-Hydroxynicotinic acid | C6H5NO3 | 139.0269 | 3.06 | 2792 |
| Linoleic acid | C18H32O2 | 280.2402 | 12.13 | 979 |
| L-Phenylalanine | C9H11NO2 | 165.0790 | 1.88 | 77564 |
| Isochlorogenic acid A | C25H24O12 | 516.1268 | 4.25 | 4354 |
| Corticosterone | C21H30O4 | 346.2144 | 6.08 | 6326 |
| Pyridoxamine | C8H12N2O2 | 168.0899 | 1.36 | 362 |
| D-Lactic acid | C3H6O3 | 90.0317 | 0.79 | 33446 |
| Arabinose | C5H10O5 | 150.0528 | 0.68 | 11849 |
| Allantoin | C4H6N4O3 | 158.0440 | 0.70 | 9743 |
| Notoginsenoside Ft1 | C47H80O17H | 962.5450 | 5.54 | 819 |
| D-Galactose | C6H12O6 | 180.0634 | 0.68 | 62256 |
| Gluconic acid | C6H12O7 | 196.0583 | 0.64 | 130560 |
| Malonic acid | C3H4O4 | 104.0110 | 0.72 | 51053 |
| Levulinic acid | C5H8O3 | 116.0473 | 1.14 | 5034 |
| Lys | C6H14N2O2 | 146.1055 | 0.61 | 2895 |
| Schaftoside | C26H28O14 | 564.1479 | 3.60 | 5527 |
| Cryptochlorogenic acid | C16H18O9 | 354.0951 | 2.42 | 26359 |
| Cholic acid | C24H40O5 | 408.2876 | 5.03 | 795 |
| D-Xylose | C5H10O5 | 150.0528 | 0.68 | 11849 |
| 3-Oxocholic acid | C24H38O5 | 406.2719 | 6.40 | 434 |
| Cyphostemmin B | C28H22O6 | 454.1416 | 4.85 | 4484 |
| Verapamil | C27H38N2O4 | 454.2832 | 5.08 | 879 |
| Ginkgolide C | C20H24O11 | 440.1319 | 4.95 | 660 |
| Phytolaccagenin +HCOOH | C31H48O7 | 578.3455 | 4.12 | 313 |
| D-Fructose | C6H12O6 | 180.0634 | 0.68 | 62256 |
| Hydroxypropionic acid | C3H6O3 | 90.0317 | 0.79 | 33446 |
| L-Sorbose | C6H12O6 | 180.0634 | 0.68 | 62256 |
| Polydatin | C20H22O8 | 390.1315 | 2.23 | 4771 |
| Hydroxyphenyllactic acid | C9H10O4 | 182.0579 | 2.44 | 3992 |
| Schisantherin E | C30H34O9 | 538.2203 | 4.74 | 20520 |
| D-Xylulose | C5H10O5 | 150.0528 | 0.68 | 11849 |
| Leonuride | C15H24O9 | 348.1420 | 4.27 | 6036 |
| Geniposidic acid | C16H22O10 | 374.1213 | 3.43 | 12026 |
| Notoginsenoside Fe | C47H80O17 | 962.5450 | 5.54 | 819 |
| Phthalic acid | C8H6O4 | 166.0266 | 3.04 | 7671 |
| Hirsuteine | C22H26N2O3 | 366.1943 | 3.69 | 8102 |
| Obacunone | C26H30O7 | 454.1992 | 3.97 | 4086 |

**Supplementary Table S2.** Mass spectrometry data and elemental composition of compounds in rat serum by UPLC-Q/TOF-MS analysis

| **Name** | **Formula** | **Mass (Da)** | **RT (min)** | **Area** |
| --- | --- | --- | --- | --- |
| Positive ion mode | |  |  |  |
| Creatinine | C4H7N3O | 113.0589 | 1.02 | 32797 |
| Cytidine | C9H13N3O5 | 243.0855 | 1.06 | 5942 |
| Leu | C6H13NO2 | 131.0946 | 1.17 | 177899 |
| Isoleucine | C6H13NO2 | 131.0946 | 1.17 | 177899 |
| Hippuric acid | C9H9NO3 | 179.0582 | 3.14 | 21284 |
| L-Tryptophan | C11H12N2O2 | 204.0899 | 2.50 | 223908 |
| 1-Methylguanine | C6H7N5O | 165.0651 | 1.96 | 8160 |
| L-Phenylalanine | C9H11NO2 | 165.0790 | 1.96 | 193057 |
| Tyr | C9H11NO3 | 181.0739 | 1.13 | 100863 |
| Niacinamide | C6H6N2O | 122.0480 | 1.69 | 50731 |
| Diosgenin | C27H42O3 | 414.3134 | 7.72 | 8571 |
| Sodium glycodeoxycholate | C26H42NNaO5 | 471.2961 | 7.76 | 377 |
| Xanthosine | C10H12N4O6 | 284.0757 | 1.89 | 844 |
| Kynurenine | C10H12N2O3 | 208.0848 | 1.96 | 5387 |
| 5''-Methylthioadenosine | C11H15N5O3S | 297.0896 | 2.84 | 6852 |
| 7-Methylguanosine | C11H15N5O5 | 297.1073 | 2.84 | 1078 |
| Deoxycytidine | C9H13N3O4 | 227.0906 | 1.13 | 21178 |
| Indoxyl | C8H7NO | 133.0528 | 3.60 | 3508 |
| Thr | C4H9NO3 | 119.0582 | 1.96 | 5517 |
| Berberine | C20H17NO4 | 335.1158 | 4.95 | 2944 |
| Hesperetin | C16H14O6 | 302.0790 | 4.83 | 5187 |
| Indole | C8H7N | 117.0578 | 2.50 | 10162 |
| Taurodeoxycholic acid | C26H45NO6S | 499.2968 | 6.37 | 1617 |
| Indoleacetic acid | C10H9NO2 | 175.0633 | 4.75 | 941 |
| Pantothenic acid | C9H17NO5 | 219.1107 | 2.05 | 19133 |
| 3-Indolepropionic acid | C11H11NO2 | 189.0790 | 5.49 | 5764 |
| Aminoadipic acid | C6H11NO4 | 161.0688 | 0.73 | 305 |
| L-Carnitine | C7H15NO3 | 161.1052 | 0.69 | 28838 |
| L-Norleucine | C6H13NO2 | 131.0946 | 1.17 | 177899 |
| Adenosine | C10H13N5O4 | 267.0967 | 1.96 | 349 |
| 4-Pyridoxic acid | C8H9NO4 | 183.0532 | 1.84 | 2054 |
| Taurocholic acid | C26H45NO7S | 515.2917 | 6.45 | 1570 |
| Phenylacetylglycine | C10H11NO3 | 193.0739 | 3.53 | 7270 |
| Ambroxane | C16H28O | 236.2140 | 11.84 | 3230 |
| Indoleacrylic acid | C11H9NO2 | 187.0633 | 2.50 | 409681 |
| Alloisoleucine | C6H13NO2 | 131.0946 | 1.17 | 177899 |
| Serotonin | C10H12N2O | 176.0950 | 1.63 | 4068 |
| Thymine | C5H6N2O2 | 126.0429 | 2.13 | 3691 |
| Wogonin 7-O-glucuronide | C22H20O11 | 460.1006 | 3.75 | 441 |
| L-Valine | C5H11NO2 | 117.0790 | 0.72 | 10517 |
| Homo-L-arginine | C7H16N4O2 | 188.1273 | 1.03 | 750 |
| Pyrrolidonecarboxylic acid | C5H7NO3 | 129.0426 | 2.16 | 1322 |
| Pyroglutamic acid | C5H7NO3 | 129.0426 | 2.16 | 1322 |
| Arginine | C6H14N4O2 | 174.1117 | 0.99 | 19977 |
| L-Methionine | C5H11NO2S | 149.0511 | 1.06 | 25533 |
| Ornithine | C5H12N2O2 | 132.0899 | 1.18 | 9251 |
| trans-Cinnamic acid | C9H8O2 | 148.0524 | 1.97 | 6860 |
| Quinaldic acid | C10H7NO2 | 173.0477 | 3.00 | 2341 |
| Cinnamic acid | C9H8O2 | 148.0524 | 1.97 | 6860 |
| o-Tyrosine | C9H11NO3 | 181.0739 | 1.13 | 100863 |
| Ethyl 4-hydroxybenzoate | C9H10O3 | 166.0630 | 8.59 | 3370 |
| Adenine | C5H5N5 | 135.0545 | 0.70 | 3826 |
| Curcumenpl | C15H22O2 | 234.1620 | 11.99 | 1775 |
| Stigmasterol | C29H48O | 412.6950 | 6.37 | 2095 |
| Thiamine Monophosphate | C12H17N4O4PS | 344.0708 | 1.07 | 616 |
| Cytosine | C4H5N3O | 111.0433 | 1.13 | 84951 |
| Kaurenoic acid | C20H30O2 | 302.2246 | 10.05 | 334650 |
| 2-Pyrrolidinone | C4H7NO | 85.0528 | 1.49 | 7343 |
| Muscone | C16H30O | 238.2297 | 12.78 | 2096 |
| 13-cis-Retinoic acid | C20H28O2 | 300.2089 | 9.47 | 2688 |
| Negative ion mode | |  |  |  |
| 7a-Hydroxy-3-oxo-5b-cholanoic acid | C24H38O4 | 390.2770 | 7.90 | 4536 |
| Aminocaproic acid | C6H13NO2 | 131.0946 | 1.22 | 172993 |
| Beta-Leucine | C6H13NO2 | 131.0946 | 1.22 | 172993 |
| Chenodeoxycholic acid | C24H40O4 | 392.2927 | 7.66 | 52300 |
| Cholic acid | C24H40O5 | 408.2876 | 7.45 | 281334 |
| Deoxycholic acid | C24H40O4 | 392.2927 | 7.66 | 52300 |
| Elaidic acid | C18H34O2 | 282.2559 | 13.09 | 107476 |
| Hippuric acid | C9H9NO3 | 179.0582 | 3.15 | 200956 |
| L-Alloisoleucine | C6H13NO2 | 131.0946 | 1.22 | 172993 |
| Linoleic acid | C18H32O2 | 280.2402 | 12.12 | 78276 |
| Ile | C6H13NO2 | 131.0946 | 1.22 | 172994 |
| Leu | C6H13NO2 | 131.0946 | 1.22 | 172994 |
| Myristic acid | C14H28O2 | 228.2089 | 11.60 | 1737 |
| Nutriacholic acid | C24H38O4 | 390.2770 | 7.90 | 4536 |
| Octadecanedioic acid | C18H34O4 | 314.2457 | 10.07 | 3845 |
| Oleic acid | C18H34O2 | 282.2559 | 13.09 | 107476 |
| Pyroglutamic acid | C5H7NO3 | 129.0426 | 0.73 | 12643 |
| Taurocholic acid | C26H45NO7S | 515.2917 | 6.45 | 44388 |
| Taurodeoxycholic acid | C26H45NO6S | 499.2968 | 6.37 | 23646 |
| Urea | CH4N2O | 60.0324 | 1.13 | 3550 |
| Ursodeoxycholic Acid | C24H40O4 | 392.2927 | 7.66 | 52300 |
| Vaccenic acid | C18H34O2 | 282.2559 | 13.09 | 107476 |
| Blinin +HCOOH | C22H32O6.HCOOH | 438.2254 | 2.05 | 864 |
| L-Tryptophan | C11H12N2O2 | 204.0899 | 2.51 | 195097 |
| Taurine | C2H7NO3S | 125.0147 | 0.67 | 9049 |
| Hyodeoxycholic acid | C24H40O4 | 392.2927 | 7.66 | 52300 |
| Hydroxyisocaproic acid | C6H12O3 | 132.0786 | 3.37 | 4800 |
| Tyr | C9H11NO3 | 181.0739 | 1.17 | 118684 |
| Hexadecanedioic acid | C16H30O4 | 286.2144 | 8.95 | 1730 |
| Pantothenic acid | C9H17NO5 | 219.1107 | 2.05 | 37560 |
| Hydroxyphenyllactic acid | C9H10O4 | 182.0579 | 2.30 | 9075 |
| Tectoridin | C22H22O11 | 462.1162 | 5.70 | 835 |
| Phe | C9H11NO2 | 165.0790 | 1.96 | 144510 |
| L-Phenylalanine | C9H11NO2 | 165.0790 | 1.96 | 144510 |
| L-Threonine | C4H9NO3 | 119.0582 | 0.68 | 2565 |
| 4-Hydroxybenzoic acid | C7H6O3 | 138.0317 | 4.43 | 655 |
| Citric acid | C6H8O7 | 192.0270 | 0.70 | 1426 |
| Scopoletin | C10H8O4 | 192.0423 | 0.69 | 158 |
| 4-Pyridoxic acid | C8H9NO4 | 183.0532 | 1.85 | 5592 |
| 4'-Hydroxyacetophenone | C8H8O2 | 136.0524 | 4.01 | 3054 |
| Indoleacrylic acid | C11H9NO2 | 187.0633 | 5.25 | 5658 |
| Kynurenine | C10H12N2O3 | 208.0848 | 1.96 | 1417 |
| Salicylic acid | C7H6O3 | 138.0317 | 4.43 | 655 |
| FAD | C27H33N9O15P2 | 785.1572 | 3.04 | 207 |
| Glycoursodeoxycholic acid | C26H43NO5 | 449.3141 | 6.61 | 26988 |
| Ornithine | C5H12N2O2 | 132.0899 | 1.22 | 10805 |
| 2,3-Diaminopropionic acid | C3H8N2O2 | 104.0586 | 1.13 | 15670 |
| 2-Hydroxybutyric acid | C4H8O3 | 104.0473 | 1.13 | 78013 |
| Pimelic acid | C7H12O4 | 160.0736 | 3.08 | 3019 |
| Leucinic acid | C6H12O3 | 132.0786 | 3.37 | 4800 |
| 3-Methyladipic acid | C7H12O4 | 160.0736 | 3.08 | 3019 |
| Azelaic acid | C9H16O4 | 188.1049 | 4.58 | 4711 |
| Methylsuccinic acid | C5H8O4 | 132.0423 | 1.39 | 1916 |
| Wogonin 7-O-glucuronide | C22H20O11 | 460.1006 | 3.74 | 716 |
| Glutaric acid | C5H8O4 | 132.0423 | 1.39 | 1916 |
| N-Formyl-L-methionine | C6H11NO3S | 177.0460 | 2.15 | 21366 |
| Indoleacetic acid | C10H9NO2 | 175.0633 | 4.74 | 725 |
| 5-hydroxytryptophan | C11H12N2O3 | 220.0848 | 2.51 | 1395 |
| Xanthurenic acid | C10H7NO4 | 205.0375 | 2.61 | 477 |
| 2,4-Dihydroxybenzoic acid | C7H6O4 | 154.0266 | 3.87 | 2563 |
| Hydroxytyrosol | C8H10O3 | 154.0630 | 3.91 | 369 |
| 2,6-Dihydroxybenzoic acid | C7H6O4 | 154.0266 | 3.87 | 2563 |
| Dipsacoside B | C17H26O11.HCOOH | 452.1530 | 6.56 | 57 |
| Thr | C4H9NO3 | 119.0582 | 0.68 | 2565 |
| 4-Hydroxycoumarin | C9H6O3 | 162.0317 | 4.74 | 125 |
| Dodecanedioic acid | C12H22O4 | 230.1518 | 6.43 | 1866 |
| Dimethylmalonic acid | C5H8O4 | 132.0423 | 1.39 | 1916 |
| Ganoderol A | C30H46O2 | 438.3498 | 7.68 | 11 |
| Indole | C8H7N | 117.0578 | 2.51 | 6852 |
| Ruscogenin | C27H42O4 | 430.3083 | 9.72 | 9833 |
| Pentadecanoic acid | C15H30O2 | 242.2246 | 12.16 | 573 |
| D-Lactic acid | C3H6O3 | 90.0317 | 0.76 | 330844 |
| L-Glutamine | C5H10N2O3 | 146.0691 | 0.67 | 7139 |
| Lys | C6H14N2O2 | 146.1055 | 0.60 | 3462 |
